# An Overview of the Factors Related to *Leishmania* Vaccine Development

**DOI:** 10.3390/vaccines14010054

**Published:** 2026-01-01

**Authors:** Luiz Felipe Domingues Passero, Italo Novais Cavallone, Gabriela Venicia Araújo Flores, Sarah Santos de Lima Melchert, Márcia Dalastra Laurenti

**Affiliations:** 1 Institute of Biosciences, São Paulo State University (UNESP), Praça Infante Dom Henrique, s/n, São Vicente 11330-900, Brazil; id.cavallone@gmail.com (I.N.C.); gabyaraflo@gmail.com (G.V.A.F.); sarahkslima@gmail.com (S.S.d.L.M.); 2 Institute for Advanced Studies of Ocean (IEAMAR), São Paulo State University (UNESP), Rua João Francisco Bensdorp, 1178, São Vicente 11350-011, Brazil; 3 Laboratory of Pathology of Infectious Diseases (LIM50), Department of Pathology, Medical School, São Paulo University, São Paulo 01246-903, Brazil

**Keywords:** leishmaniasis, clinical forms, vaccine, immunity

## Abstract

Leishmaniasis is an infectious disease caused by several species of *Leishmania* parasites that preferentially infect macrophages as host cells. These intracellular parasites can evade the main microbicidal effector mechanisms of phagocytic cells and, in turn, are able to stimulate marked Th2 or regulatory T cell immune responses, which are not protective for the host. The presence of a non-protective immune response, together with the multiplication and spread of *Leishmania* parasites throughout the tissues, leads to the main clinical forms of leishmaniasis, such as cutaneous and visceral leishmaniasis. Although some clinical forms can be reproduced in experimental hosts such as mice and hamsters, these models do not fully mimic natural infection, which, in fact, impacts experimental vaccine development. For example, BALB/c mice are generally infected with around one million parasites, whereas humans are not infected with more than 1000 parasites, together with vector saliva. This excessive number of parasites in experimental models may affect the efficacy of vaccines in preclinical studies. Indeed, many experimental studies conducted over the past 20 years have shown only partial protection, regardless of the vaccine generation, host species employed, or the use of adjuvants. This review aims to summarize the main aspects associated with *Leishmania* vaccine development, including parasite diversity, host factors, immune responses, adjuvants, and antigens. Although many elegant studies have been conducted, it is possible that some essential step is still missing for the development of an effective vaccine for human use.

## 1. Introduction

Leishmaniasis is a group of zoonotic diseases that affect humans and several species of wild and domestic animals. It is caused by a digenetic protozoan belonging to the Trypanosomatidae family, genus *Leishmania*. Its biological cycle is carried out in two different hosts, one vertebrate and the other invertebrate. The vertebrate hosts include a wide variety of mammals, including rodents, edentates, marsupials, canines, and primates, including humans. Invertebrate hosts are small insects belonging to the order Diptera, family Psychodidae, subfamily Phlebotominae, and the two main genera *Lutzomyia* (New World) and *Phlebotomus* (Old World).

Leishmaniasis is endemic on five continents, in 98 countries located in tropical and subtropical regions. Currently, more than 1 billion people live in endemic areas of leishmaniasis and are at risk of contracting infection. An estimated 30,000 new cases of visceral leishmaniasis (VL) and more than 1 million new cases of cutaneous leishmaniasis (CL) occur annually [1]. Climate change, as well as the socioeconomic changes resulting from the globalization process, not only make it difficult to control the disease but also increase the number of people affected. A very striking example is the clear process of urbanization of leishmaniasis, a process closely related to rural exodus, unemployment, expansion of slums, wars, among others.

Considering the expansion of the disease, to date, there are no effective control measures such as vaccination, and the therapeutic arsenal is limited and shows, in addition to undesirable adverse reactions, reports of resistance [2]. Despite this, the development of safe and effective immune prophylactic measures should be a priority in leishmaniasis; in fact, considering all immunogens characterized and studied in several experimental models so far, why have we not yet discovered a significant and potent vaccine? To answer this question, it is very important to discuss some aspects of pathogenesis, parasite diversity, the type of vaccines and adjuvants employed so far in the context of leishmania vaccine development. Therefore, this review intends to discuss the main aspects associated with vaccine development, as well as all the significant details related to the parasite and host relationship.

## 2. Immune Response in Leishmaniasis

To understand the way how vaccines work in leishmaniasis, it is imperative to understand how the host immune response to *Leishmania* determines the fate of this parasite. The inoculation of promastigote forms by the insect vector into the vertebrate host triggers defense mechanisms related to the innate and acquired immune response.

The innate immune response begins with the action of the complement system, which has been widely studied. Antagonistic functions are attributed to the complement system: the lytic capacity of extracellular parasites and the favoring of the intracellular survival of the protozoan by aiding the phagocytosis of *Leishmania* sp. by macrophages, via the interaction between C3bi fragment and complement receptors found on the surface of these cells. However, the action of the complement system relies on the parasite species and on the size and composition of the lipophosphoglycan (LPG) molecule in the metacyclic parasite [3]. Furthermore, parasites can also evade the complement by the action of leishmanial protein kinase (LPK-1), which phosphorylates complement components [4].

Polymorphonuclear leukocytes are important in parasite phagocytosis. Experimental in vivo studies have shown that neutrophils are the first cells to migrate to the parasite inoculation site, resulting in parasite degeneration [4]. However, subsequent studies have shown that neutrophils would be temporary host cells for *Leishmania* until these infected cells undergo apoptosis. During this event, macrophages phagocytosed these infected cells without triggering the microbicidal mechanisms, allowing a silent entry of the parasites into these professional phagocytic cells, establishing infection [5].

With respect to the main antigen-presenting cells (APC), the macrophages and dendritic cells are one of the most important ones. In macrophages, the main host cell for *Leishmania*, the interaction between promastigote forms and the host cell involves a ligand-receptor contact, followed by a series of biochemical reactions that can lead to the activation or inhibition of the microbicidal functions of the host cell, which will determine the fate of the parasite in the host [6]. Concerning dendritic cells, studies on the role of Langerhans cells in the immunopathogenesis of cutaneous leishmaniasis caused by *L. major* in BALB/c mice have shown that, contrary to the assumption about the importance of these cells in the development of the cellular immune response, it has been demonstrated that dendritic cells from the dermis are the responsible for the antigen-specific stimulation of T cells [7]. Subsequent experiments showed that Langerhans cells could process and present parasitic antigens through the major histocompatibility complex II (MHCII) to CD4^+^ T cells, which differentiate into regulatory T cells (Treg), which will control an excessive immune response against *Leishmania*. Furthermore, this cell subset can produce interleukin-10 (IL-10) and tumor growth factor β (TGF-β), which directly inhibit the effector activity and proliferation of effector T cells, impacting a proper activation of macrophage, allowing the spreading and persistence of *Leishmania* at the site of infection [8,9]. These data suggest that Langerhans cells could inhibit inflammatory events in *Leishmania* infection [9]. In human localized cutaneous leishmaniasis caused by *L.* (*Viannia*) *braziliensis*, whose patients showed strongly positive DTH for the leishmanial antigen, the density of Langerhans cells in the inflammatory infiltrate was smaller compared to lesions caused by *L.* (*Leishmania*) *amazonensis*, whose patients presented less expressive or even absent DTH. The study also showed a negative correlation between the density of Langerhans cells and the density of CD4^+^ and CD8^+^ T lymphocytes in the clinical and immunopathological spectrum of American cutaneous leishmaniasis [10]. These data suggest that subsets of dendritic cells exert different immunomodulatory responses in the host during *Leishmania* infection. Furthermore, it has been observed that NK cells can also trigger apoptosis in infected cells, or even modulate their activity by producing IL-12 cytokine [11].

During the interaction between antigen-presenting cells and T lymphocytes, a chronic immune response will be induced and will influence the course or progression of the disease. In general, if APCs are able to differentiate T-CD4^+^ Th1 from naïve T cells, a high production of TNF-α and IFN-γ cytokines will be produced that would be able of activating macrophage, culminating in the elimination of the intracellular parasites [12]. In contrast, if antigen-presenting cells stimulate the differentiation of T-CD4^+^ Th2 cells, the parasite will reproduce, causing active lesions, since this T lymphocyte subset produces IL-4 and IL-13 cytokines, which do not activate macrophages, allowing the spread of amastigote forms. Furthermore, IL-4 aids the development of B lymphocytes, which produce and secrete antibodies, without protective effect to the host [13]. Among T lymphocytes, the presence of CD8^+^ T cells in the tissues infected by *Leishmania* sp. has been associated with healing and protection of human and murine leishmaniasis through their cytotoxic function, as well as IFN-γ production, a potent inducer of nitric oxide, which promotes the destruction of parasites [14,15].

**Figure 1:**
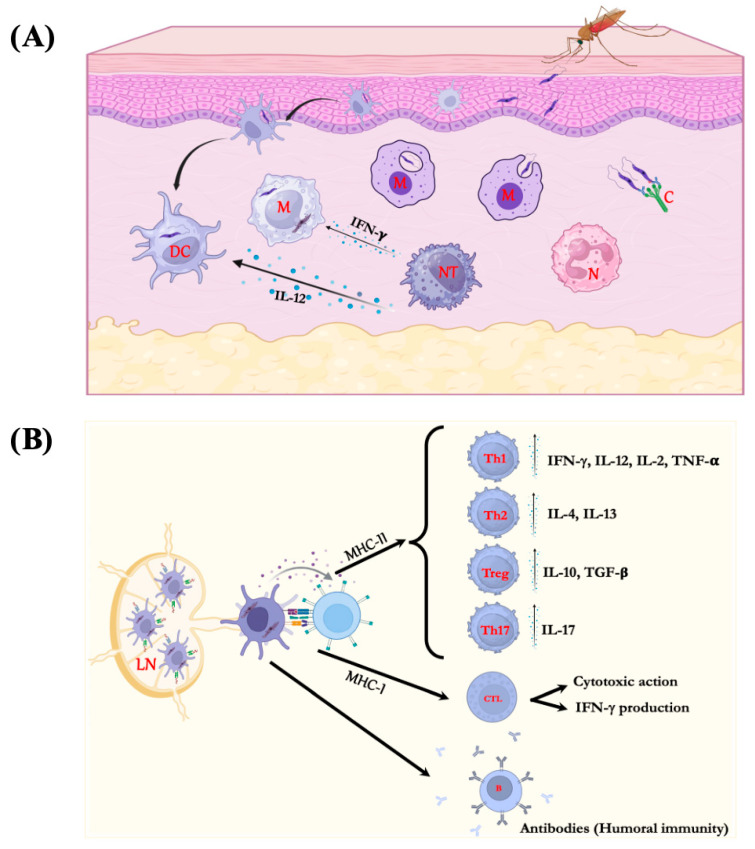
Diagram showing in (**A**) nonspecific inflammatory response to infection by parasites of the genus *Leishmania*, involving innate immune cells such as dendritic cells (DC), macrophages (M), neutrophils (N), natural killer cells (NT), and the complement system proteins (C). In (**B**) the activation of specific immunity in regional immune organs such as lymph nodes (LN) can be observed with antigen presentation by the MHC and specific lymphocyte stimulation, such as: T helper 1 (Th1) with production of pro-inflammatory cytokines (IFN-γ, IL-2, IL-12, TNF-α), T helper 2 with production of anti-inflammatory cytokines (IL-4, IL-13), T regulatory cells with production of regulatory cytokines (IL-10, TGF-β) and T cytotoxic (TCL) with cytotoxic action or IFN-γ production. B lymphocytes (**B**) can also be activated for antibody production.

Another subset of effector T cells is Th17, which produces IL-17 cytokine and exhibits a distinct effector function from Th1 or Th2 cells in leishmaniasis. The primary function of Th17 cells is to eliminate microorganisms through the production of IL-17, a pro-inflammatory cytokine that acts on a wide range of cell types to induce the expression of IL-6, IL-8, GM-CSF (granulocyte-macrophage colony-stimulating factor), G-CSF (granulocyte colony-stimulating factor), and metalloproteases; IL-17 is also a key cytokine to active and recruit neutrophils to the site of infection [16]. The role of Th17 cells in leishmaniasis is controversial, whereas, in cutaneous and mucocutaneous leishmaniasis, they were associated with tissue damage and pathology, but protection in visceral leishmaniasis [17,18]. Figure 1 summarizes the immune response triggered by *Leishmania* parasites.

Although Figure 1 summarizes the immune response of the host to *Leishmania* sp., it is still important to mention that differences may exist, considering the biodiversity of the hosts as well as the parasites. Furthermore, other factors, such as the saliva vector, gender, and physiological status of the hosts, can modify this prototype immune response [19,20,21].

## 3. Experimental Models

Experimental *in vivo* infection remains the optimal model for characterizing the disease and its impact on the vertebrate host. The selection of an appropriate model is predicated on two fundamental criteria: its physiological similarity to humans and its ease of handling. The most frequently utilized animal models are rodents, predominantly hamsters and mice, as they exhibit high reproductive capacity in captivity, require minimal housing and maintenance conditions, are easily handled, and are susceptible to nearly all species of *Leishmania*. To establish an experimental model for leishmaniasis, there are several key criteria to consider, such as genetic background of the vertebrate host, the sex of the animal, the growth phase of the parasite in culture, as well as the size of the inoculum, the route of inoculation, the site, and the method of parasite delivery. These criteria are imperative in elucidating the function of immune cells, cytokines, and effector mechanisms in regulating *Leishmania* parasites as well as progression or progression of the disease, as in the case of vaccination.

### 3.1. Experimental Models in Visceral Leishmaniasis

The dog (*Canis familiaris*) is considered a significant domestic reservoir of *Leishmania* (*Leishmania*) *infantum*, playing a key role in human infection by serving as a source of parasites to the phlebotomine sand fly vectors, which transmit VL [22]. Experimental infections using *L.* (*L.*) *infantum* provide a valuable laboratory model for studying VL, as they closely replicate the natural progression of the disease. In both dogs and humans, infections caused by the *Leishmania* (*L.*) *donovani* complex are often subclinical; however, without appropriate treatment, they can progress to severe visceral disease, potentially resulting in death. The clinical manifestations are similar in both species, typically beginning with an asymptomatic phase that advances to symptoms such as weight loss, anemia, lymphadenopathy, and fever. One notable difference is the presence of skin lesions in dogs, which are not observed in human cases [23,24,25].

Dogs are significant not only due to their epidemiological role as natural reservoirs but also to their significance as experimental models in VL research, particularly because of the similarities in disease progression between canine and human infections. In this context, Maia and collaborators reported that 75% of Beagle dogs intravenously infected with *L.* (*L.*) *infantum* amastigotes exhibited symptoms, including lymphadenomegaly and mild skin lesions. The spleen showed the highest parasite load, and *Leishmania* DNA was detected in the skin of all dogs, even in the absence of visible lesions. The authors suggest that intravenous infection with amastigotes facilitates rapid and consistent disease progression, making this model useful for evaluating therapies and vaccines. Furthermore, the detection of *Leishmania* in the skin of asymptomatic (experimentally infected) dogs highlights their potential role in parasite transmission to sand fly vectors [26], which was further reinforced in naturally infected dogs from endemic areas [27]. However, the use of dogs as an experimental model for VL presents several limitations, including high costs, complex maintenance, restrictions on animal numbers per experiment, and ethical considerations. Furthermore, these studies also point out some differences between the human and canine VL. Due to these constraints, rodent models are currently preferred in most experimental studies.

To overcome the issues associated with canine experimental leishmaniasis, golden hamsters and mice have been employed in the study of VL. The available results suggest that the mouse model develops a subclinical and limited infection, making this model comparable to self-controlled oligosymptomatic cases and therefore useful for studying the protective immune response [23,24]. In contrast, the experimental model of VL using the golden hamsters, *Mesocricetus auratus*, reproduces human disease [28]. In studies focused on viscerotropic *Leishmania* species, the clinicopathologic characteristics and immunopathologic mechanisms of visceral leishmaniasis (VL) in hamsters bear a striking resemblance to those observed in humans, while markedly differing from those found in murine models [28,29]. Upon systemic infection, hamsters exhibit an increased parasite load within the viscera, along with progressive cachexia, hepatosplenomegaly, pancytopenia, hypergammaglobulinemia, and, sometimes, death [30]. Although some degree of infection control is evident in the liver due to the formation of granulomas, the infection in the spleen tends to evolve progressively and chronically, leading to intense macrophagic parasitism and the disruption of the structure of white pulp with the replacement of lymphocyte cellularity by red pulp cells containing parasitized macrophages and plasma cells [31,32]. Although the hamster develops clinical-pathological characteristics and immunopathological mechanisms similar to human VL, it is important to note that it is not a perfect model; many hamsters develop ascites, renal failure, and nephrotic syndrome, conditions that are only rarely observed in humans and canine animals [29]. Nevertheless, the use of hamsters remains constrained due to a paucity of reagents (antibodies, cell markers, and cytokines) with defined specificity that are available for the study of the role of immune response in the pathology in this model.

The use of non-human primates is due to their phylogenetic proximity, anatomical and physiological similarity, and immune response resemblance to humans, making them attractive models for evaluating the pathogenicity of infectious diseases, as well as for the development of vaccines and therapies [33]. The use of the rhesus macaque (*Macaca mulatta*) seems to be an interesting experimental model for visceral leishmaniasis caused by *L.* (*L.*) *infantum* [34]. On the other hand, it was demonstrated that *Cebus apella* monkeys infected with *L.* (*L.*) *infantum chagasi* (syn. *L.* (*L.*) *infantum*) develop a transient infection, as these animals can mount an effective cellular immune response that controls the spread of *L.* (*L.*) *infantum*. According to the authors, the rhesus monkey is not a recommended model for mimicking a natural infection caused by viscerotropic *Leishmania* species. Non-human primates, as mentioned above, are the animals of choice for safety, immunogenicity, and protection studies in phase III clinical trials of vaccines and drug candidates for VL. However, they are more difficult to use due to high maintenance and experimental costs, strong ethical considerations, and the fact that some species are endangered. Furthermore, as observed in several studies, phylogenetic proximity does not guarantee that it is the best model for VL. Table 1 summarizes the studies that standardized models of VL.

### 3.2. Experimental Models in Cutaneous Leishmaniasis

The *Leishmania* species responsible for cutaneous leishmaniasis causes infections with different patterns of progression in animal models. In general, *L*. (*L*.) *mexicana* and *L*. (*L*.) *amazonensis* have shown high virulence in hamsters and BALB/c mice [41]. In contrast, species such as *L*. (*V*.) *braziliensis* and *L*. (*V*.) *panamensis* induce lower virulence in rodent models [42,43]. These data suggest that the differences between the evolution of infection in experimental models may limit the direct applicability of experimental data [44].

In humans, infections caused by *Leishmania* (*L.*) *amazonensis* exhibit a broad spectrum of clinical manifestations, ranging from localized cutaneous to mucocutaneous and diffuse cutaneous forms [45]. Infection with *L*. (*L*.) *amazonensis* and *L*. (*L*.) *mexicana* is associated with different disease patterns in mice, with some strains, such as C57BL/6 or C3H, developing chronic disease, whereas BALB/c and C57BL/10J produce progressive ulcerative lesions with cutaneous metastasis [46]. In the acute phase of *L*. (*L*.) *amazonensis* infection, susceptible mice (C57BL/10 and CBA) and relatively resistant mice (DBA/2) both exhibit predominant infiltration of eosinophils and mast cells. However, susceptible mice show higher numbers of amastigotes, increased tissue destruction, and a stronger humoral immune response. In resistant mice, parasite persistence is associated with infection of dendritic cells and fibroblasts [47]. The susceptibility of C57BL/10 mice to *L*. (*L*.) *amazonensis* is related to the absence of a Th1-type cellular immune response. This differs from BALB/c mice, where susceptibility is associated with a Th2-type response [48].

In rhesus monkeys (*Macaca mulatta*) infected with *L*. (*L*.) *amazonensis*, initial erythematous and papular skin lesions have been observed that progress to nodules that ulcerate before regressing and healing. Parasites are detected in biopsies during the initial phase of the nodular lesions. The authors suggest that the progression and resolution of *L*. (*L*.) *amazonensis* infection in rhesus monkeys is very similar to that observed in humans and can be used to elucidate the mechanisms of protective immunity in cutaneous leishmaniasis and to evaluate the efficacy of new vaccines [49]. Resolution has also been observed in *Sapajus apella* monkeys infected with *L*. (*L*.) *amazonensis*. However, *L*. (*V*.) *braziliensis* persisted with a low number of amastigote forms in the skin of *S. apella* primates. These results demonstrate that differences in parasite species and their immunomodulatory properties in vertebrate host cells influence the outcome of infection, as demonstrated by infection with *L*. (*L*.) *amazonensis* and *L*. (*V*.) *braziliensis*, which induce a different infection profile in the non-human primate *S*. *apella*, as observed in humans [50].

Table 2 summarizes the experimental models of cutaneous leishmaniasis standardized in recent years.

While experimental models have significantly advanced our understanding of leishmaniasis, their intrinsic limitations must also be carefully considered. Notably, no single model fully replicates the complex clinical variability observed in humans, which is shaped by factors such as host genetics, immune responses, nutrition, and co-infections. In addition, standard experimental conditions, such as high inoculum doses and the absence of sandfly saliva, differ markedly from natural transmission, potentially influencing disease progression. Furthermore, larger animal models like dogs and non-human primates present practical constraints related to high costs, logistical complexity, and ethical considerations.

In summary, animal models continue to represent valuable tools for investigating the biology of *Leishmania* infection and for supporting the development of effective therapies. However, careful consideration is necessary when translating experimental findings to human disease. Refinement of these models, through strategies that more closely resemble natural transmission, such as low-dose inoculation and inclusion of vector saliva, may improve their translational relevance and applicability to clinical settings.

## 4. Adjuvants

### 4.1. Description of Adjuvants

Vaccines act through the recognition of antigens by the immune system, triggering humoral and cellular responses that enhance the host’s resistance to infection [52]. Although extensive efforts have been devoted to developing vaccines against leishmaniasis, and some have been commercialized for canine visceral leishmaniasis, no vaccine is yet available for the anthroponotic form of the disease. This limitation likely arises from the inability of parasitic antigens to elicit strong cellular immunity and lasting immunological memory, which can be improved by using an appropriate adjuvant system [53].

Adjuvants are compounds of synthetic or natural origin, ranging from simple molecules to complex particulate systems, capable of functioning as immunostimulants or antigen delivery platforms [54]. They enhance immunogenicity primarily by improving antigen retention at the injection site and facilitating its uptake by immune cells. This process recruits immune cells, enables controlled antigen release, and prolongs macrophage interaction, thereby strengthening the immune response [54]. Additionally, adjuvants can activate and mature antigen-presenting cells (APCs), promote cytokine production, stimulate inflammasomes, and induce local inflammation and cell recruitment [55].

Through these mechanisms, adjuvants can reduce the required antigen dose and number of immunizations, counteract immune senescence, extend vaccine half-life, and increase antigen stability [56]. In summary, antigen–adjuvant combinations are highly effective: while antigens direct the adaptive immune response toward a specific pathogen, adjuvants activate the innate immune system via pattern recognition receptors (PRRs) that recognize pathogen-associated molecular patterns (PAMPs) [57]. In the following sections, it will be performed a brief description of the most used adjuvants.

### 4.2. Alum Adjuvant

**Figure 2:**
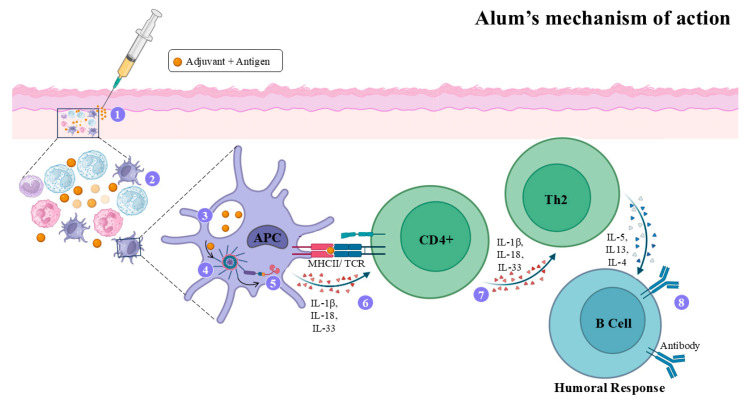
Alum’s mechanism of action. Antigens and adjuvants are slowly released into the tissue (1), recruiting innate immune cells to the site of immunization (2). Dendritic cells phagocytosis and process the antigen, presenting it to CD4^+^ T lymphocytes through MHCII molecules (3). At the same time, the adjuvant stimulates the endogenous immune response through the activation of the NLRP3 protein (4), which binds to ASC and pro-caspase 1, and caspase 1, assembling the NLRP3 inflammasome (5), which promotes the secretion of pro-inflammatory cytokines such as IL-1β, IL-18, and IL-33 (6). These cytokines drive the differentiation of CD4^+^ T cells into the Th2 subset (7), stimulating the production of antibodies by B cells (8).

Alum forms a depot at the injection site, ensuring a slow and sustained antigen release that prolongs immune activation, enhancing antigen uptake by APCs at the depot site [50]. The inflammatory process that takes place in the dermis stimulates B lymphocytes to produce antibodies [51]. Figure 2 depicts the action mechanism of this adjuvant.

In leishmaniasis, it has been observed that alum has a limited capacity to elicit Th1 responses, restricting its applicability. Vaccines formulated with alum combined with soluble *Leishmania* antigen (SLA) failed to enhance protection in experimental VL. Moreover, high IL-4 levels were detected in animals receiving adjuvant alone, suggesting the intrinsic capability of alum at inducing B cell differentiation [58]. In contrast, immunization with *Leishmania* antigens plus *Lutzomyia longipalpis* peptide maxadilan (FL-MAX) combined with alum induced a more robust protection in mice, associated with lower parasite burden, reduced footpad swelling, and production of antibodies recognizing the P1, P2, and P11 regions of MAX. Nevertheless, FL-MAX caused prolonged inflammation, persisting for over 240 days post-injection [59]. Overall, alum’s efficacy in leishmaniasis is limited, as it preferentially induces a Th2 response dominated by IL-4, IL-5, and IL-10 production, an immune profile ineffective against obligate intracellular pathogens, as discussed above. However, alum’s performance improves when combined with immunostimulatory adjuvants such as BCG, which promote stronger Th1-type responses. Since alum does not activate TLRs, the inclusion of TLR agonists like BCG represents a promising strategy to enhance its immunostimulatory potential [60]; however, such a strategy makes a prototype vaccine very expensive.

### 4.3. Freund’s Adjuvant (FA)

Freund’s adjuvant is composed of the Complete Freund’s Adjuvant (CFA), containing heat-killed mycobacteria (*Mycobacterium tuberculosis* or *Mycobacterium butyricum*), and (2) Incomplete Freund’s Adjuvant (IFA), which lacks mycobacterial components [56]. Freund’s adjuvants generally consist of an emulsifying detergent, responsible for stabilizing antigen-containing aqueous droplets, and a mineral oil phase that slows absorption due to its hydrophobic nature. This configuration results in the gradual uptake of droplets by macrophages and prolonged antigen exposure, eliciting both Th1 (CFA) and Th2 (IFA) immune responses [61].

In CFA, the inclusion of mycobacteria introduces pathogen-associated molecular patterns (PAMPs) that activate pattern recognition receptors (PRRs), enhancing APC activity and promoting the release of pro-inflammatory cytokines such as TNF-α, IL-12, and IL-6 [62]. IL-12 further recruits NK cells, which produce IFN-γ, amplifying antigen presentation and driving Th1 differentiation [63]. Consequently, CFA induces both a depot effect and strong innate immune activation, resulting in a pronounced delayed-type hypersensitivity (DTH) response, including against self-antigens in autoimmune models [62]. By contrast, IFA, which lacks mycobacterial components, provides weaker innate activation but maintains a slow-release depot effect that favors antibody-mediated responses with a predominant Th2 profile [64,65].

**Figure 3:**
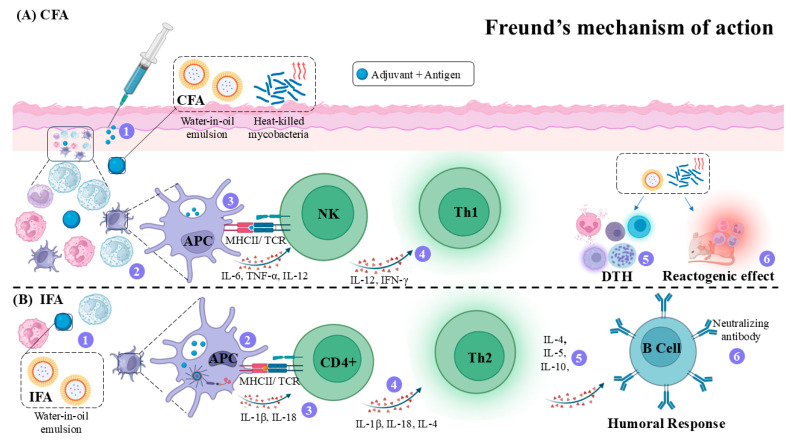
The mechanism of action of CFA (**A**) begins with the deposition of heat-killed mycobacteria in a *w*/*o* emulsion (1), which, when injected, recruits immune cells to the site of immunization (2). The antigen and adjuvant are processed by APCs, which release pro-inflammatory cytokines (IL-6, TNF-α, and IL-12), recruiting NK cells (3). These cells produce IL-12 and IFN-γ, stimulating the differentiation of Th1 lymphocytes (4). The *w*/*o* emulsion with paraffin and mycobacteria prolongs the Th1 response, leading to DTH (5), inflammatory and toxic effects to the host (6). In IFA (**B**), deposition of the adjuvant with antigen recruits immune cells (1), which are processed by APCs (2). The absence of mycobacteria in the adjuvant results in the production of cytokines IL-1β and IL-18, stimulating CD4^+^ T lymphocytes (3). These cells produce IL-4, IL-1β, and IL-18, promoting the maturation of the Th2 cell subset (4). Th2 cell subset, in turn, produces IL-4, IL-5, and IL-10 (5), activating B cells and inducing antibody production (6), establishing the humoral response.

In immunization protocols, CFA is commonly applied in the first dose to initiate a strong innate activation, whereas boosters employing IFA maintain immune stimulation with reduced local reactogenicity [66]. Despite their potency, CFA and IFA have limited use in humans due to adverse effects, including severe local inflammation, tissue necrosis, and potential carcinogenicity associated with the persistence of non-biodegradable mineral or paraffin oils, which may induce granulomatous lesions [67]. The detailed action mechanism of this adjuvant is illustrated in Figure 3.

In leishmaniasis, it was observed that BALB/c mice immunized three times with recombinant thiol-specific antioxidant (TSA) formulated with different adjuvants (IFA, alum + BCG, or Montanide) presented similar lymphocyte proliferation, IFN-γ production, and IgG1/IgG2a levels after challenge with *L. major* [68]. A similar result was obtained when IFA was formulated with total *L. braziliensis* antigen; however, its efficacy was similar to that of other adjuvants [69]. These data suggest that FA can be replaced by other adjuvants, considering the side effects it induces in the host. Furthermore, comparative studies already reinforced that other formulations, such as the liposomal encapsulation of antigens, exhibit higher adjuvant properties than FA [70]. Conversely, some studies reported limited vaccine efficacy in *L. donovani* models when the antigens lipophosphoglycan, polyacrylic acid, and a DNA vaccine (pVAX-P1) were formulated with FA, since immunized animals showed low survival (~66%), modest parasite-load reduction (38–65.8%), and failure to elicit a strong Th1 response [71,72].

Overall, the literature reveals heterogeneous results regarding CFA and IFA efficacy in experimental vaccines against *Leishmania*. While some studies demonstrate protective responses, others report partial or negligible efficacy, likely reflecting differences in adjuvant composition and mechanisms. CFA induces strong Th1 and Th17 responses through mycobacterial components that activate NOD2/RIP2/IRF5 pathways, generating robust IFN-γ responses essential for parasite control [62,63]. However, its intense inflammatory profile may limit consistent protection across models. In contrast, IFA, lacking mycobacteria, generally promotes weaker innate activation and a Th2-biased or mixed response, which may be less effective against leishmaniasis.

### 4.4. Bacillus Calmette–Guérin (BCG) Adjuvant

**Figure 4:**
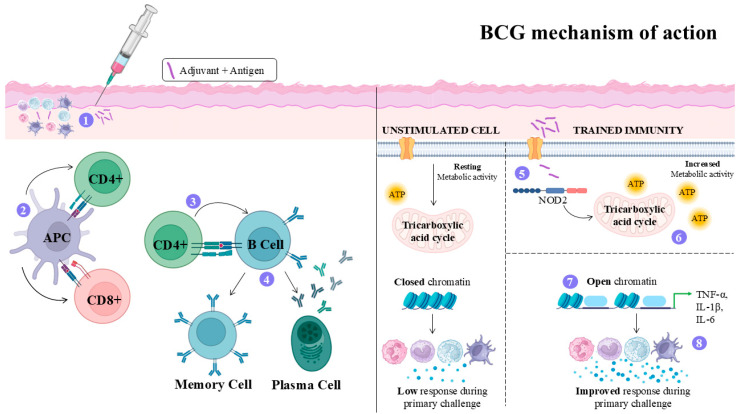
BCG adjuvant leads to the activation of macrophages, neutrophils, and dendritic cells (1), presenting antigens to CD4^+^ and CD8^+^ T lymphocytes (2), which lead to an adaptive immune response. CD4^+^ T lymphocytes stimulate B lymphocytes (3), which produce memory and plasma cells (4). In addition, BCG adjuvant induces metabolic reprogramming that increases glycolysis, by the signalization of NOD2 (5), providing the energy needed for cellular activation (6). Concomitantly, it induces epigenetic reprogramming in innate immune cells through the opening of chromatin in promoter regions (7), leading to the high expression of pro-inflammatory cytokines (8).

Bacillus Calmette–Guérin (BCG) is a live attenuated strain of *Mycobacterium bovis*, developed by Albert Calmette and Camille Guérin [73]. Further studies led to the development of the first oral vaccine ƒto prevent disseminated forms of tuberculosis in children, including meningitis and miliary tuberculosis. It later became one of the most widely used vaccines globally [74]. The cellular and molecular changes that BCG induces in the host were explored in Figure 4.

The use of BCG as a vaccine adjuvant against leishmaniasis has been shown to enhance pro-inflammatory cytokine production in models challenged with *L. major*, *L. infantum*, and *L. donovani*. BCG functions as a TLR agonist, promoting the recognition of PAMPs and, when combined with specific antigens, strengthening antigen presentation and overall immune activation, particularly through TLR2, TLR4, and TLR9 pathways [75]. In murine (Swiss albino and BALB/c mice) and hamster models of leishmaniasis, the immunization with recombinant dipeptidylcarboxypeptidase protein (rLdDCP) from *L. donovani*; recombinant cysteine proteinase (rLdccys1) from *L.* (*L.*) *chagasi*; killed *L. major* vaccine (KLV); autoclaved *L. major* (ALM) vaccine; and *L.* (*L.*) *donovani* antigens (triosephosphate isomerase and enolase) plus BCG adjuvant caused an increase in IFN-γ, IL-12, and NO in comparison with animals non-immunized controls and animals immunized with the antigen alone, suggesting that BCG enhanced the Th1 response and consequently reduced parasite burden in target organs of animals challenged with *L. major*, *L. infantum*, or *L. donovani* [76,77,78,79,80], suggesting that this adjuvant may be incorporated in experimental vaccines.

### 4.5. Monophosphoryl Lipid A (MPL) Adjuvant

**Figure 5:**
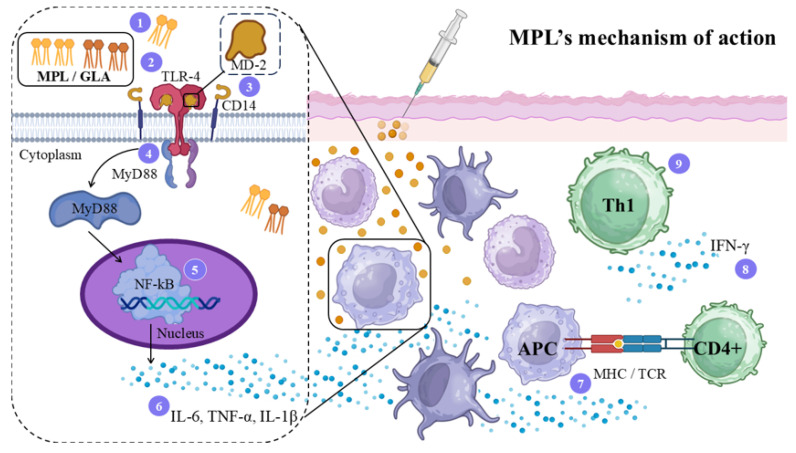
MPL or GLA is injected as a vaccine adjuvant (1). The adjuvant particles stimulate TLR-4 expressed on the cell membrane (2), which is activated by the coreceptors CD14 and MD-2 (3), recruiting the MyD88 protein (4). The signaling cascade activates NF-kB (5), initiating the expression of pro-inflammatory cytokines such as IL-1β and IL-6, and TNF-α (6). The production of pro-inflammatory cytokines and the action of CD14 stimulate the processing of antigens and their presentation to the MHC (7), which results in the maturation of APCs, as well as the activation of IFN-γ-producing CD4+ T lymphocytes (8), which leads to polarization towards Th1 (9).

The main natural ligand of TLR4 is lipopolysaccharide (LPS), composed of a glycan polymer, an oligosaccharide, and lipid A, the latter being essential for receptor activation due to the specific interaction with MD-2 [81]. Based on this structure, several synthetic TLR4 agonists were developed, such as monophosphoryl lipid A (MPL) and glucopyranosyl lipid A (GLA) [82]. MPL induces a systemic immune response and favors the activation of the Th1 immune response [83]. Among TLR agonists, it is the only adjuvant licensed and commercially available for use in vaccines, including the VHB vaccine Fendrix, the HPV vaccine Cervarix, and for use in patients with renal insufficiency, Fendrix vaccine being considered safe and non-toxic [84,85]. Figure 5 illustrates the action mechanism of this adjuvant.

In a comparative study, Thakur et al. assessed the prophylactic effect of killed *L. donovani* antigens (KLD) formulated with alum, saponin, cationic liposomes, or MPL in BALB/c mice. Although killed antigen preparations typically show limited efficacy [86], the addition of cationic liposomes or MPL substantially enhanced protection to 83.4–92.8% and 86–93.7%, respectively, and enhanced a Th1 immune response [86]. A similar response was observed when hamsters were immunized with the recombinant antigens rA6 or rF14 in the presence of MPL adjuvant in visceral leishmaniasis [87].

The recombinant polyprotein rLeish-111f, composed of TSA, LmSTI1, and LeIF antigens, was formulated with MPL-SE. Following three subcutaneous immunizations, mice developed mixed IgG1/IgG2a responses, high IFN-γ levels, and protection to *L. major* challenge comparable or superior to soluble leishmanial lysate [88]. Subsequently, Fujiwara et al. formulated the same experimental vaccine in the presence of MPL-SE or AdjuPrime that was administered subcutaneously in three doses at four-week intervals in dogs. Both adjuvants induced high IgG2 titers, but the vaccine built with MPL-SE triggered a markedly stronger Th1 response, with an IgG2/IgG1 ratio ~40-fold higher than AdjuPrime, suggesting superior protective potential against canine visceral leishmaniasis [89]. However, MPL-SE failed to provide efficacy in a phase III trial of the Leish-111f vaccine in dogs challenged with *L. infantum*, where more than 87% of vaccinated animals developed disease [90].

In humans with mucocutaneous leishmaniasis receiving sodium stibogluconate treatment, adjunct immunization with LEISH-F1 formulated in MPL-SE (three doses at 28-day intervals) was safe, well-tolerated, and immunogenic. Vaccination increased LEISH-F1-specific IL-2-producing memory CD4^+^ T cells, a response associated with clinical cure, supporting the safety and immunogenicity of LEISH-F1/MPL-SE in patients [91].

### 4.6. CpG-Oligodeoxynucleotides (CpG ODN) Adjuvant

CpG ODNs are short synthetic single-stranded DNA molecules that mimic bacterial DNA and function as immunomodulatory adjuvants by activating TLR9 within endocytic vesicles of plasmacytoid dendritic cells (pDCs) and B cells, thereby initiating innate and adaptive immune responses [92], resulting in the recruitment of macrophages, NK cells, and T lymphocytes to the immunization site [93].

**Figure 6:**
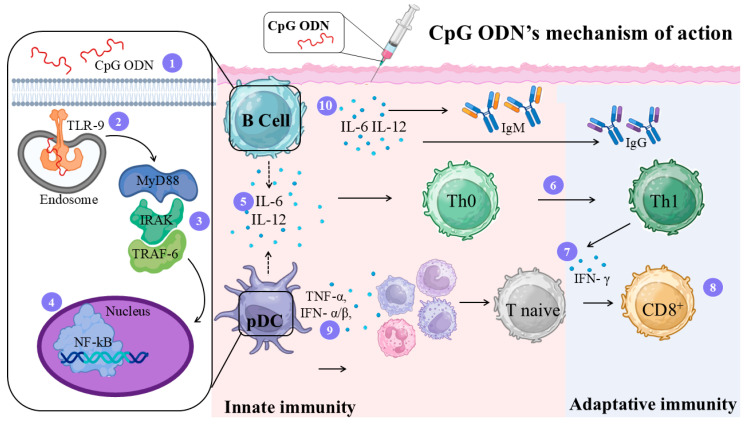
CpG ODN is phagocytosed by B cells and pDCs (1), processed and recognized in the endosome by TLR-9 (2). TLR-9 stimulation leads to a cascade of MyD88, IRAK, and TRAF-6 signaling (3), which activates NF-κB (4), producing proinflammatory cytokines (5), which lead to the maturation of naïve lymphocytes to the Th1 profile (6), and the secretion of IFN-γ (7). IFN-γ production by CD4^+^ Th1 lymphocytes leads to the activation of antigen-specific CD8^+^ T cells (8). The pDCs express TNF-α, IFN-ꞵ, and IFN-α, activating and recruiting NK, monocytes, and neutrophils (9). Activated B cells increase the production of IL-6 and IL-12, and secrete the immunoglobulins IgM and IgG (10).

Despite their overall Th1-polarizing activity, CpG ODNs encompass at least three classes with distinct immunological profiles [94]. Type B (K) CpGs primarily activate B cells and antigen-presenting cells, inducing IL-6 and IL-12 production and stimulating Th1 differentiation with robust IgG2a and IgM responses but minimal IFN-α and IFN-γ induction [93]. Type A CpGs, in contrast, strongly induce IFN-α secretion and promote the maturation of APCs and pDCs [95]. Type C CpGs combine features of both classes, simultaneously stimulating IL-6 production by B cells and IFN-α secretion by pDCs [93]. The molecular and cellular mechanisms that CpG induces in the host are illustrated in Figure 6.

Different experimental approaches have evaluated CpG ODN as an adjuvant in *Leishmania* vaccine formulations. When combined with soluble *L. major* antigen (SLA), CpG ODN markedly increased anti-*Leishmania* IgG2a and IFN-γ levels in BALB/c mice compared with SLA alone [96]. Subsequent studies demonstrated that incorporating CpG ODN into delivery systems further strengthened its immunostimulatory effect. Tafaghodi and colleagues investigated CpG ODN in combination with autoclaved *L. major* (ALM) antigens encapsulated in poly(d,l-lactide-co-glycolide) (PLGA) nanospheres or alginate (ALG) microspheres. In both systems, BALB/c mice receiving ALM + CpG ODN, either free or encapsulated, exhibited higher IgG2a/IgG1 ratios, increased IFN-γ production, and reduced IL-4 levels relative to ALM alone or infected controls [97]. These findings confirmed that CpG ODN promotes a Th1-biased response and that encapsulation strategies can prolong and amplify this protection.

The adjuvant effect of CpG ODN was also validated using recombinant antigens. Its association with SODB1 and Pxn4 reduced lesion size and parasite burden in BALB/c mice challenged with *L. donovani*, outperforming recombinant antigens alone [98]. Likewise, CpG ODN formulated with recombinant *L. major* STI1 (rLmSTI1), either free or in liposomes, induced smaller lesions, lower splenic parasite loads, and higher IgG2a responses compared with non-adjuvanted groups, consistent with a CpG-driven Th1 profile [99].

Overall, these studies highlight CpG ODN as a promising adjuvant capable of eliciting strong and protective Th1-type immunity in *Leishmania* models. However, its activity is species-dependent, robust in mice due to broader TLR9 expression, but more restricted in humans, where TLR9 is limited to plasmacytoid dendritic cells and select B-cell subsets [100]. Thus, large-animal models may provide an essential translational bridge for advancing CpG-based vaccine formulations toward clinical development [101].

Adjuvants play an essential role in amplifying immune responses to antigens; however, conventional adjuvants often display limited immunostimulatory capacity, underscoring the need for next-generation adjuvants. An ideal adjuvant should be safe, stable before administration, biodegradable, and easily eliminated while promoting a targeted and effective immune response and remaining cost-effective for large-scale production. Advancing research on the mechanisms and immunological properties of various adjuvants is fundamental for rational vaccine design, enabling the development of more effective and personalized immunization strategies.

## 5. Vaccines for Leishmaniasis—20 Years of Studies on the First, Second, and Third-Generation Vaccines

Studies of vaccination employ different platforms to produce vaccines, in leishmaniasis some of them have pointed out the significance of second- and third-generation vaccines in inducing effective and long-lasting immunity over live or first-generation vaccines. In fact, the COVID-2019 pandemic also reinforced this statement, by showing the World an important breakthrough in human medical history, which a global vaccination with mRNA vaccines, viral vectors, or protein subunit vaccines avoided around 20 million deaths [102]. In fact, the global emergence was huge, allowing a fast and reliable process of vaccine production given the availability of the virus strains, viral genome, and identification of the conserved spike protein. Furthermore, mRNA vaccines, non-replicating viral vectors, and lipid nanoparticle systems was already recognized as a safe strategy to be employed in different medical purposes. Although pandemics brought irreparable losses worldwide, it also gave us significant lessons: (1) global collaboration in science is essential; (2) it is imperative to obtain as much as possible information about the genome and relationship of infectious agents and hosts; (3) different vaccine platforms allow a rapid development of vaccines and (4) vaccines are safe to humans. Unfortunately, in neglected diseases, although substantial efforts have been made by the scientists, few candidates have been approved to be translated into human health, as is the case of the vaccine RTS,S/AS01 (Mosquirix) and R21/Matrix-M, both approved by the World Health Organization and used to protect humans from malaria [103]. The vaccines cited above are the first ones approved in the context of parasitic infections, even inducing partial protection. In fact, this experience with malaria vaccine brought the idea that it is possible to build and develop a vaccine for neglected diseases even in the context of parasites with complex life cycles and different interactions with humans, vectors, wild or peri-domestic animals, as is the case with leishmaniasis. In this specific situation, this task should be significantly difficult due to the existence of a diversity of parasite species causing a spectrum of human diseases, which in turn, makes it hard to obtain a single vaccine able to confer protection across most pathogenic species. The following sections will discuss some vaccine platforms and the main findings on the experimental vaccines developed in the last 20 years. Articles were selected after a bibliographic survey using a Boolean search, performed in the Scopus and PubMed databases, during November 2024 to March 2025 and the combination of words was used to expand the possibility of finding data that would meet the expectations of the present study: “(vaccine OR vaccination OR immunization OR leishmanization)” AND (first-generation vaccine OR whole cell lysate OR total antigen) AND (second-generation vaccine OR subunit vaccine OR protein) AND (third-generation vaccine * OR DNA vaccine OR RNA vaccine)”.

### 5.1. Leishmanization and the Generations of Vaccines Against Leishmaniasis

In the context of vaccination, some authors consider live vaccines as the first generation [104]; we have chosen to include them in the topic of leishmanization, discussed in the next paragraph.

Leishmanization refers to the first prophylactic attempts against leishmaniasis, which involve the inoculation of live parasites in less exposed areas of the body to induce immunity against more severe subsequent infections [105]. Studies on leishmanization began in 1940 and continued until the 1980s, using strains of *L. tropica* [98,106,107] and *L. major* [108]. Despite demonstrating protection against reinfections in humans, issues such as parasite persistence, long-lasting and non-self-healing wounds, need for treatment in some cases, hypersensitivity reactions, and lack of standardization were reported [98,106,107,108], preventing further development and prompting the search for safer vaccines [109].

During the past 20 years, some studies have maintained the principles of leishmanization while modifying the method to produce a safer vaccine. In this method, Laabs and colleagues [110] immunized C57BL/6 intradermally with 10^4^ live *L. major* promastigote forms plus CpG DNA adjuvant. This immunization protocol triggered IL-2 production by dendritic cells and IFN-γ by NK cells. Furthermore, vaccinated animals did not develop skin or mucosal lesions; however, no subsequent challenge was performed [110]. In an earlier study by the same group [111], it was observed that 100% of mice vaccinated exhibited protection against a subsequent challenge (15 weeks after vaccination) with 500 metacyclic promastigotes (an estimated concentration of a natural sandfly inoculation) [111].

Leishmanization studies employing *L. tarentolae*, a non-pathogenic species for humans, have been reported [112,113,114]. BALB/c mice were vaccinated intraperitoneally with 5 × 10^6^ stationary-phase promastigotes of *L. tarentolae* in a single dose, and after 6 weeks post-vaccination, these animals exhibited cross-protection after challenge with 5 × 10^7^ promastigotes of *L. donovani*, since these animals exhibited 80% less parasites in the spleen and liver than non-vaccinated mice [113]. The immunogenic and protective potentials of *L. tarentolae* were highlighted in another study, in which BALB/c mice were vaccinated intraperitoneally with 5 × 10^6^ recombinant *L. tarentolae* expressing the A2 gene of *L. donovani* (a virulence factor expressed in amastigotes of virulent *Leishmania* strains) in a single dose [112]. Six weeks after immunization, animals received an inoculum of 10^7^ *L. infantum* intravenously. Vaccinated animals exhibited 90% less parasites in the spleen and liver, which was associated with the elevation of IFN-γ levels and reduction in IL-5, suggesting that this vaccine protects animals by increasing the Th1 immune response [112]. A more recent study demonstrated that the immunization with a single dose containing 2 × 10^5^ live *L. tarentolae* promastigotes with CpG ODN protected BALB/c mice from the challenge with 2 × 10^5^ *L. major* metacyclic promastigotes [114].

Another approach consists of the use of live attenuated strains of *Leishmania*. The attainment of the result can be achieved in several ways, such as culture in media supplemented with antibiotics such as gentamicin [115], genetic modifications for drug susceptibility (e.g., Ganciclovir and 5-fluorocytosine) [116], deletion of several genes such as HSP70-II [117] and centrin [109,118,119]. Furthermore, the use of naturally attenuated strains such as Sri Lankan *L. donovani*, which does not cause visceral disease [120], is another interesting possibility. A study that followed dogs for 24 months after a subcutaneous single-dose vaccination with 5 × 10^7^ gentamicin-attenuated *L. infantum* stationary-phase promastigotes demonstrated that 100% of the vaccinated group showed no leishmanial DNA, and 97.8% of animals exhibited no clinical signs of the disease [115], this protection was associated with higher levels of IgG2 compared to the IgG1 levels found in the sera of vaccinated animals, as IgG2 antibodies are associated with asymptomatic infection, whereas IgG1 is related to disease progression [115].

Davoudi and collaborators studied the protective potential of subcutaneous leishmanization in C57BL/6 mice using 2 × 10^6^ promastigotes of *L. major* (*Lmtkcd*^+/+^), which were susceptible to Ganciclovir and 5-fluorocytosine due to the inclusion of thymidine kinase (*tk*) and cytosine deaminase (*cd*) genes in the genome of this parasite, respectively [116]. The enzyme encoded by the *tk* gene targets ganciclovir, a nucleoside analog that blocks DNA replication, while the enzyme encoded by the *cd* gene acts on the 5-fluorocytosine molecule, generating the active drug metabolite that inhibits RNA synthesis [121]. The mice were treated with both drugs on day 8 post-vaccination to attenuate the drug-sensitive strain and, after 3 weeks, C57BL/6 mice were challenged with wild-type *L. major* promastigotes; studies on lesion size and tissue parasitism showed that vaccinated animals did not develop lesions and that the parasite burden in the spleen was reduced by 90% in comparison to the non-vaccinated mice [116]. Another study showed that the leishmanization in BALB/c mice employing HSP70-II knockout *L. infantum* promastigotes provided more than 90% cross-protection against an infection with 10^3^ *L. major* metacyclic promastigotes, as measured by the splenic parasite load [117]. In another study, it was observed that intradermal vaccination with centrin-deleted *L. major* stationary-phase promastigotes (*Lmcen*^−/−^) offered 90% reduction in the spleen and liver parasite loads in hamsters subsequently infected (seven weeks later) with *L. donovani* promastigotes or exposed to infected sandflies [118].

Leishmanization represents the historical foundation for the development of vaccines against leishmaniasis, and current studies employing this methodology in murine and canine models have enriched the literature regarding the understanding of protective immunity against the parasites. It is notable that leishmanization using non-pathogenic, attenuated strains or those associated with Th1 immune-stimulating adjuvants has achieved remarkable results in reducing parasite burden and providing protection against infection challenges. However, the parasite is not completely neutralized or eliminated, rendering these vaccines neither effective nor safe. The demand for a more humanized immunization approach and the reported evidence of cross-immunity (the ability of immunization with one strain to confer protection against infection by different *Leishmania* strains) have encouraged investigations into the subsequent three generations of vaccines. Table 3 summarizes the main data on leishmanization.

### 5.2. First Generation of Leishmania Vaccines

The first generation of vaccines consists of killed parasite antigens, which can be obtained by different chemical methods, such as the use of formalin and merthiolate [123,124], or physical methods, such as sonication, autoclaving, freezing and thawing, and pasteurization [125]. This class of vaccines does not bring significant concerns regarding the emergence of clinical signs and symptoms, transforming the vaccinated individual into a reservoir, allowing the transmission of parasites by a vector or even a possible reactivation of the pathogenic state of the attenuated *Leishmania* strains used in protocols of leishmanization [126,127].

Current studies with first-generation vaccines use various methods for obtaining total antigens, different adjuvants, and different hosts, such as rodents [123,124,125,128], dogs [129,130,131], and humans [132]. Experimentally, a vaccine produced with the whole cell lysate of *L. amazonensis* promastigote forms was tested with different preservative agents, such as thimerosal and phenol [123]. Two groups of C57BL/10 mice were vaccinated with these vaccines (100 mg/dose) plus *Corynebacterium parvum* as adjuvant (250 mg/dose) in two subcutaneous doses. This adjuvant was used due to its ability to activate both cellular and humoral immune responses [133]. Seven days after the last immunization, the animals were challenged with *L. amazonensis* promastigotes. Both vaccines conferred 50% protection (defined by the appearance of lesions); however, animals immunized with the phenol-preserved vaccine developed smaller wounds than those seen in animals immunized with the thimerosal-preserved vaccine; in addition, among vaccinated animals that developed lesions, they were ten times smaller compared to those observed in control groups [123].

Germanó and collaborators (2020) evaluated different methodologies to obtain *L. amazonensis* first-generation vaccines, such as autoclaving, freeze-thawing, pasteurization, and sonication [125]. These experimental vaccines were combined with the adjuvant polyinosinic:polycytidylic acid [(Poly (I:C)], a TLR3 agonist [125]. Once formulated, BALB/c mice were immunized with three doses of these vaccines (100 µg/dose of *L.* (*L.*) *amazonensis* extracts plus 50 µg of Poly (I:C) by the subcutaneous route, animals were challenged with *L.* (*L.*) *amazonensis* promastigotes [125]. The group of animals vaccinated with the sonicated formulation associated with poly (I:C) adjuvant exhibited the smallest lesion size as well as less splenic involvement. Furthermore, such animals produced the highest levels of anti-*Leishmania* IgG1 and IgG2a isotype in comparison to the other first-generation vaccines, it was observed that the IgG2a/IgG1 ratio was higher [125]. This vaccine formulation stimulated a classical Th1-type response, but the increase in IL-4 levels and the production of basal levels of IL-10 may have contributed to the persistence of parasites in vaccinated animals [125].

The immunogenic and protective potential of *L.* (*V.*) *shawi* amastigote and promastigote forms was also analyzed as first-generation vaccines [134]. BALB/c mice received the whole cell lysates of amastigote or promastigote forms (25 μg/dose) subcutaneously twice at a regular interval of seven days. After the challenge with *L. shawi* promastigotes, it was observed that animals immunized with promastigote whole cell lysate developed smaller lesions and exhibited 70% less skin parasitism compared with non-immunized animals. The partial protection was associated with the elevation of IFN-γ, IL-12, and TGF-β cytokines. In contrast, immunization with amastigote forms whole cell lysate exacerbated the infection, increasing lesion size by 50% and skin parasite burden by 450% compared with non-immunized animals, this response occurred due to the high levels of TGF-β and low of IL-2, IFN-γ, and nitric oxide produced by mononuclear cells [134]. These findings highlight that antigens derived from amastigote forms show immunosuppressive properties, while those from promastigote forms have an immunostimulatory potential in the host.

First-generation vaccines have already been studied in combination with saponin adjuvant [129,130] and saliva gland extract from *Leishmania* vectors. In this sense, two experimental vaccines were formulated, the first one with the whole antigen of *L.* (*V.*) *braziliensis* (LbSap) [129] plus saponin, and another constituted with *L.* (*V.*) *braziliensis* whole antigen plus salivary gland extract (SGE) of *Lutzomyia longipalpis* and saponin (LbSapSal) [130]. These vaccines were administered subcutaneously to dogs in a three-dose regimen at four-week intervals, each dose [129,130]. Both vaccines induced a cross humoral immune response with anti-*Leishmania chagasi* antibodies IgG1 and IgG2, while vaccination with LbSapSal also developed anti-SGE IgG1 and IgG2; both immunizations stimulated the proliferation of B, T CD4^+^ and CD8^+^ T lymphocytes [129,130]. Furthermore, it was observed that increased CD4^+^ and CD8^+^ T cell populations were correlated with asymptomatic animals showing low parasite burden [129,130,135]. Both vaccines stimulated MHC-I expression, and in in vitro experiments using PBMCs showed elevated NO production in the supernatants of cells from vaccinated animals stimulated with soluble antigens of *L. braziliensis* and *L. chagasi* [129,130]. Thus, these vaccines exhibited strong antigenic potential and could activate an immune response associated with the resistance pole; however, further studies on cytokine profiling are necessary to better understand the T cell response elicited by this type of immunogen in dogs [129,130].

A formalin-killed *L. major* promastigotes vaccine (FKP) was formulated with several adjuvants, such as Montanide ISA 720 [136], Bacille Calmette–Guerin (BCG), and Alum [124]. BALB/c mice were subcutaneously vaccinated with three doses of this vaccine and then challenged with *L. major* promastigote forms [124]. Among all vaccines tested, the one formulated with the Montanide ISA 720 adjuvant generated the highest IFN-γ production, the lowest parasite load (96% reduction), and the smallest lesion size (80% reduction) compared to the control animals. In contrast, animals vaccinated with FKP without any adjuvant exhibited intermediate lesion sizes, lower IFN-γ production, and a higher parasite burden compared to the other vaccinated groups [124]. These studies highlight the importance of using adjuvants to provide greater protection in different experimental models.

The combination of antigenic extracts from different species of *Leishmania* has also been reported during the last 20 years of vaccine research [131]. Giunchetti and collaborators developed a vaccine containing *L. amazonensis* and *L. braziliensis* whole parasite lysates plus BCG as an adjuvant. This vaccine was assessed in beagle dogs by administering three subcutaneous doses at four-week intervals. It was observed that vaccination employing *L.* (*L.*) *amazonensis* and *L.* (*V.*) *braziliensis* whole parasite lysates, especially after the second and third immunizations, caused a significant increase in IgG anti-*Leishmania* antibodies. Furthermore, it was observed that these antibodies could recognize *L. chagasi* antigens [131], suggesting that this experimental vaccine would be able to induce cross-protection. Subsequently, Grenfell et al. developed two vaccines, one containing crude extracts of *L. braziliensis* promastigotes plus saponin (50 µg) and the second one was constituted by *L. amazonensis* promastigotes and saponin; both vaccines were administered in groups of BALB/c mice in three subcutaneous doses at one-week intervals [128]. In this study, the main objective was to investigate whether such vaccines were able to confer cross-protection after challenge with *L. chagasi* promastigotes. In fact, after challenge with infective *L. chagasi*, the immunized animals presented a reduction of 25% and 50% in the hepatic and splenic parasite loads, respectively. Furthermore, a significant inhibition of IL-4 and IL-10 production was observed [128]. The reactivity of anti-*L. amazonensis* antibodies to *L. chagasi* antigens indicate an analogous antigenic repertoire between these species, allowing for an increased spectrum of vaccine protection by using these species combined in the vaccine composition [131].

Some studies have also been conducted in humans. Vélez and collaborators [132] assessed the cross-immunity of the Leishvacin^®^, composed of merthiolate-killed *L. amazonensis* in humans [132]. Three intramuscular doses (0.36 mg/mL/dose) were administered at 20-day intervals to a group of soldiers who worked in endemic areas of *L. panamensis* infection. At the end of 15 months of monitoring, it was observed that the incidence of cutaneous leishmaniasis in vaccinated and placebo groups was similar (7.8% and 6.8%, respectively), concluding that the vaccine, despite being safe, did not provide protection against *L. panamensis* [132]. Khalil and collaborators demonstrated that a single vaccination using killed *L. major* plus BCG in children stimulated a cellular immune response in 50% of the patients, as measured by the skin test [137]; however, the immune response decreased after 18 months of the immunization to 25%, suggesting that this immunization scheme does not induce a long-lasting immunity [137]. Possibly, the failure of this vaccination is associated with the presence of several antigens able to induce and expand Th2 lymphocyte clones, restricting the potential of first-generation vaccines [134].

Although these studies show some discrepant results on the protective effect of first-generation vaccines during experimental and natural infections, all these studies pointed out the safety profile of such vaccines in different hosts, which in turn suggests that defined antigens will also present a safe profile during immunizations. Table 4 summarizes the main findings about first-generation vaccines.

### 5.3. Second Generation of Leishmania Vaccines

Studies with first-generation vaccines allow the investigation of safer prophylactic methods than leishmanization. However, it is evident that although the total antigenic extract provides protection against subsequent challenge, not all antigens are responsible for this protection [143]. This observation enabled the development of the second generation of vaccines, which involves the isolation of a specific parasite antigen; the modification of proteins and the production of chimeras; and the use of the secretome produced by *Leishmania* in vaccination protocols [104].

Among the defined *Leishmania* antigens studied over the past 20 years are the *Leishmania*-Activated C-Kinase Antigen (LACK) [144,145]; histones [146]; hydrophilic acylated surface protein B (HASPB) [146]; the 63-kDa metalloprotease (GP63) [147]; among others. The LACK antigen, also known as the *Leishmania* homologue of receptors for activated C kinase or P36 (a 36-kDa protein), is conserved in *Leishmania* species and is expressed in both promastigote and amastigote forms [148], features that make this antigen a significant protein to be studied in immunization protocols. Stober and collaborators [144] studied the antigenic potential of a recombinant *L. major*’s LACK protein that was expressed in *Escherichia coli*. BALB/c mice were vaccinated subcutaneously with two doses of 100 µg of LACK, followed by a challenge with *L. major* promastigote [144]. It was verified that immunized animals produced high levels of IFN-γ; however, the LACK vaccine failed to protect against infection, possibly due to increased IL-10 production [144]. More recently, it was demonstrated that LACK from *L. donovani* in the recombinant form also fails to induce protection in visceral experimental leishmaniasis [144]. Although LACK is a conserved antigen among *Leishmania* species and is expressed in all stages of the parasite, it has no antigenic ability to induce a protective immune response, thus encouraging the search for new antigenic factors for vaccine development.

GP63, or leishmanolysin, is a glycoprotein abundantly expressed on the plasma membrane of promastigotes and at lower density on the membrane of *Leishmania* amastigotes [149]. It is a zinc-dependent metalloprotease, which enables it to cleave host proteins and thus evade the host immune response [149]. This antigen has been studied extensively in the host and parasite context, including the development of vaccines. The studies conducted by Kaur et al. focused on the formulation of vaccines containing GP63 alone (10 µg/dose) or in combination with other antigens such as heat shock protein 70 kDa (Hsp70, 10 µg/dose), total autoclaved *L. donovani* antigens (ALD, 2.5 mg/dose), or the adjuvant monophosphoryl lipid A (MPL-A) [150,151]. BALB/c mice were vaccinated with two subcutaneous doses of these vaccines; the animals were challenged intracardially with *L. donovani* promastigotes [150,151]. All vaccine formulations resulted in progressive reductions in parasite burden in the spleen and liver at the analyzed experimental time points, with the formulation GP63 + Hsp70 + MPLA providing the highest level of protection. In addition, all formulations induced the production of Th1-type cytokines (IFN-γ and IL-2) and reduced the levels of Th2 and regulatory cytokines (IL-4 and Treg). However, mice immunized with the GP63 + Hsp70 + MPLA vaccine displayed the most effective microbicidal cytokine response among all formulations. Control formulations lacking GP63 failed to induce antigenic or protective responses, highlighting the importance of developing vaccines that include the GP63 antigen.

Another study showed that a vaccine containing 2.5 µg of gp63, purified from *L. donovani* promastigotes and encapsulated in cationic liposomes, was administered intraperitoneally in three doses to BALB/c mice, which were subsequently challenged with *L. donovani* promastigotes [147]. All immunized mice survived up to three months after challenge, while 50% of non-immunized mice died; additionally, the vaccine reduced spleen and liver parasite loads by 80% compared to unvaccinated animals. Furthermore, it was observed that these vaccine formulations induced proliferation of CD4^+^ and CD8^+^ T cell along with an elevation in IFN-γ production, accounting for the partial protection observed. These studies identify GP63 as an immunogenic and protective component against infection, effective across different inoculum sizes and various formulations and delivery systems (both free and liposomal forms).

Histones are highly conserved proteins that associate with DNA to structure chromatin. Like other eukaryotic organisms, histones are present in *Leishmania* protozoa with a high degree of conservation among species [104]. HASPB is a membrane protein found in the extracellular and intracellular forms of *Leishmania* sp.; therefore, it can be a significant target to develop efficient prophylactic measures. The protective immune response potential of *L. infantum* histone and HASPB recombinant proteins was studied separately and in combination [146]. Three vaccine formulations were produced with the recombinant proteins: H1 (100 µg of histone 1), HASPB1 (100 µg of HASPB1), and H1+HASPB1 (100 µg of each protein); Montanide ISA 720 adjuvant was combined with such vaccines. The dogs were vaccinated with three intradermal doses of each formulation, and 45 days after the last immunization, the animals were challenged with *L. infantum* promastigotes. Adverse reactions were not recorded during immunizations, except for local inflammation after the second dose [146]. Following challenge, protection against the manifestation of symptoms (lymphoadenopathy, onychogryphosis, alopecia, cutaneous lesions, weight loss, or keratoconjunctivitis) was observed: HASPB1 and H1+HASPB1 protected 50% of dogs from developing leishmaniasis symptoms, while the H1 vaccine protected 62.5% of the animals, which also had the lowest parasite load in the bone marrow and lymph nodes [146]. It is important to emphasize that this protection was observed under extreme experimental conditions, in which the infection inoculum was overestimated (10^8^ parasites). The vaccines exhibited a partial protective effect in dogs, and it was observed that the vaccine composed exclusively of the H1 protein was more effective than the combination of antigens. This finding highlights the importance of antigen selection in vaccine design, as combining antigens may impair the overall immune effect.

The antigenic potential of other *L. donovani* proteins has been investigated and classified according to their molecular weights: 91 (LD91), 72 (LD72), 51 (LD51), and 31 (LD31) kDa [152] as well as a recombinant protein with 78 kDa [153]. Proteins extracted from stationary-phase *L. donovani* promastigotes were incorporated into cationic liposomes, generating four vaccines containing 2.5 µg of their respective antigens (LD91, LD72, LD51, LD31). These vaccines were administered intraperitoneally in three doses to BALB/c mice; ten days after the final dose, the animals were challenged with 2 × 10^7^ stationary-phase *L. donovani* promastigotes. The LD91 antigen provided low protection after challenge, whereas the LD72, LD51, and LD31 proteins reduced parasite loads in these organs by 60–70%. Two additional vaccines were formulated using the recombinant protein with 78 kDa: (1) 10 µg of r78 + 40 µg of MPL-A, and (2) 10 µg of r78 encapsulated in cationic liposomes. The vaccination protocol consisted of three intraperitoneal injections to BALB/c mice; fifteen days after the last dose, the animals were challenged with *L. donovani* promastigotes. The r78 protein conferred a progressive reduction in parasite burden in infected animals—from 47% (r78 + MPL-A) and 49% (r78 in liposomes) at 15 days post-challenge to 96% and 97%, respectively, at 90 days post-challenge. In addition, both r78-based vaccines stimulated IFN-γ production throughout the experimental period.

Other proteins have been studied in recent years. Vakili and colleagues produced a multi-epitope vaccine composed of four antigenic proteins: histone H1, sterol 24-C-methyltransferase (SMT), a hypothetical protein specific to *Leishmania* (LiHy), and an antigenic protein specific to *Leishmania* (LSAP) [154]. This vaccine, tested alone (30 μg of the protein) or combined with Freund’s adjuvant [30 μg of the protein in 30% (*v*/*v*)], was administered in two subcutaneous doses (2-week intervals) to BALB/c mice, followed by the challenge with *L. infantum* promastigotes. Both formulations stimulated IFN-γ production and inhibited IL-10 production, culminating in a significant reduction in the parasite load of the spleen by 80%. Lage and colleagues developed a vaccine using a protein they termed ChimeraT, produced with prohibitin, eukaryotic initiation factor 5α, and the hypothetical LiHyp1 and LiHyp2 proteins; saponin was used as an adjuvant [155]. The vaccine, composed of 15 μg of ChimeraT plus 15 μg of saponin, was administered in three subcutaneous doses to BALB/c mice, followed by a challenge with *L. infantum* promastigotes. The vaccine stimulated the production of IFN-γ before and after the infectious challenge and reduced the parasitic load by 80% in the spleen and liver; 90% of parasite reduction was recorded in the bone marrow and lymph node. In the same study, Lage and collaborators used Chimera T, its individual component antigens, and the *L. infantum* whole cell lysate to stimulate PBMCs from healthy individuals and patients (before and after treatment). It was observed that Chimera T induced higher IFN-γ production in PBMCs compared to the isolated antigens. Interestingly, Chimera T did not stimulate IL-10 production, whereas total *L. infantum* antigens reduced IFN-γ production and promoted IL-10 secretion. These results suggest that chimeric proteins are promising, as they combine two or more antigens into a single protein that the immune system can recognize, considering the broad and diverse proteome of *Leishmania* protozoa. Moreover, their ability to stimulate a Th1-type response in human PBMCs highlights their potential use in the prevention of human leishmaniasis.

The antigenic potential of proteins released by *Leishmania* sp. has been reported [156]. Proteins released by virulent *L. infantum* promastigotes were divided into two groups: those larger than 75 kDa (LiRic1) and smaller than 37 kDa (LiRic2), which were formulated into two vaccines—5 μg/mL of LiRic1 and LiRic2. BALB/c mice were immunized with three intraperitoneal doses followed by a booster dose seven weeks after the first dose; one week after the last dose, the mice were challenged with *L. infantum* promastigotes. Immunized animals showed reductions of 50% (LiRic1) and 66% (LiRic2) in splenic parasite burden. In addition, both vaccines stimulated IFN-γ production by CD4^+^ T cells when stimulated with the LiRic2 antigen. Proteins released by *L. shawi* promastigotes were separated into three antigenic fractions: >75 kDa (LsPass1), 75–50 kDa (LsPass2), and <50 kDa (LsPass3), which constituted three vaccines—25 μg of LsPass1, LsPass2, or LsPass3, respectively [157]. BALB/c mice were immunized with two intradermal doses and challenged with *L. shawi* promastigotes [157]. Mice vaccinated with LsPass2 and LsPass3 presented significant reductions in skin parasite burden of 72% and 92%, respectively, compared with non-vaccinated and infected controls. In addition, these mice exhibited a significant reduction in lesion size from the fourth week of infection onward; however, no reduction in parasite burden or lesion size was observed in animals treated with LsPass1 [157]. Although immunization with LsPass1 increased IFN-γ and TNF-α mRNA expression, cytokines considered pro-inflammatory and associated with the activation of antimicrobial responses, this vaccine also stimulated IL-10 mRNA production, which may have contributed to infection persistence and disease progression. Immunization with LsPass2 also induced IFN-γ and TNF-α mRNA expression, accompanied by low IL-10 levels, whereas LsPass3 immunization resulted in moderate IFN-γ and TNF-α production compared to the other formulations. It should be emphasized that a wide variety of proteins are released by *Leishmania* sp., and that antigenic pools may contain proteins that either stimulate or suppress the host immune response.

Some second-generation vaccines have been licensed for prophylactic use in canine leishmaniasis, including Leishmune^®^, LeishTec^®^, CaniLeish^®^, LetiFend^®^, and Leish-111f^®^ [158,159,160,161,162]. Leishmune^®^ was produced in Brazil by *Zoetis* and licensed in 2004; it was composed of the fucose-mannose ligand (FML) of *L. donovani* plus saponin as an adjuvant [163]. The first report on the efficacy of the use of FML and saponin occurred in 1992 by Palatnik-de-Souza and collaborators in which three intraperitoneal doses were administered to BALB/c mice, each composed of 150 μg of FML and 150 μg of saponin. One week after the last dose, the animals were challenged with *L. donovani* amastigotes [164]. After 15 days of challenge, it was observed that the animals exhibited 84% less parasites in the liver and a significant increase in the humoral response against the FML antigen compared to unvaccinated animals. This study prompted the subsequent formulation of Leishmune^®^, as well as the studies related to the efficacy of this vaccine during natural infection. A field study conducted by Nogueira and collaborators observed the vaccine’s protection in dogs, which received three subcutaneous doses [162]. After 11 months of vaccination, 56.7% of non-vaccinated dogs were positive for *Leishmania*, while 100% of vaccinated dogs showed no clinical signs compatible with leishmaniasis nor detectable parasites in the skin, blood, or lymph nodes. However, in another field study, conducted by Marcondes and collaborators, vaccinated dogs exhibited anti-*Leishmania* IgG levels similar to the antibody levels of naturally infected dogs. Despite such results, this study was discontinued due to pet owners’ refusal to allow further sample collection, making it impossible to determine the vaccine’s efficacy [165]. Another field study reported that 89% of vaccinated dogs remained asymptomatic and parasite-free after 11 months of vaccination, but 5.1% of vaccinated dogs transmitted the parasite to sandflies during the xenodiagnosis test, contradicting claims that Leishmune^®^ prevents vector transmission [163,166]. The production and commercialization of Leishmune^®^ was discontinued in 2014 due to a lack of evidence of its efficacy in trials.

In the study by Fernandes and collaborators, a field trial was conducted with LeishTec^®^ [166]; a vaccine composed of the recombinant A2 protein from *L. donovani* amastigotes plus saponin as adjuvant; it was produced in Brazil by *Ceva Saúde Animal* and licensed in 2007 [163]. LeishTec^®^ was administered subcutaneously in three doses, demonstrating a higher protection rate than Leishmune^®^, with 92% of vaccinated dogs remaining asymptomatic and undetectable for the parasite; however, 5.4% of vaccinated dogs tested positive for *Leishmania* transmission in xenodiagnosis [166]. Two additional field trials were conducted during 2016 [167] and 2017 [160]; both using the same three-dose subcutaneous vaccination scheme. In the first study, 63.7% of vaccinated dogs remained asymptomatic and parasite-free [167], while in the second study, the protection rate was 69% [160], but the xenodiagnosis test pointed out that 35.7% of vaccinated animals transmitted the parasite to sandflies [167]. However, similar to Leishmune^®^, LeishTec^®^ was discontinued by the Ministry of Agriculture and Livestock due to noncompliance with the minimum required antigen content [168].

Letifendi^®^, produced in Spain by *Leti Pharma*, contains the recombinant Q protein, which is composed of five antigenic factors derived from four *L. infantum* proteins [159]. These proteins are widely recognized by the sera of dogs with visceral leishmaniasis [169]. Three antigenic factors originate from ribosomal proteins (LiP0, LiP2a, and LiP2b), while the remaining two are derived from histone 2A (H2A); such proteins contain epitopes that are recognized by B cells, suggesting immunogenicity. The first reports on the protective potential of the recombinant Q vaccine were conducted in mice [170] and dogs [171], prior to the period covered by this review. In these studies, BALB/c mice vaccinated with two doses (15 days apart) of 2 μg of Q protein plus 20 μg of CpG-ODN, and subsequently challenged with *L. infantum* promastigotes, exhibited 99% reduction in parasite burden in the spleen and liver, along with increased IFN-γ production. In contrast, dogs vaccinated with three intraperitoneal doses (boosters administered 21 and 44 days after the first immunization) containing 4 μg/kg of Q protein plus BCG, and subsequently challenged with *L. infantum* promastigotes, did not develop clinical signs of disease. Furthermore, only 50% of vaccinated and infected animals showed parasites in lymph nodes at 150 days post-infection, compared with 100% positivity in non-vaccinated, infected dogs. Even before being licensed as a vaccine, a study with the Q protein was conducted using vaccination protocols with different numbers of doses [172]. In this case, it was observed that by changing the dose of Q vaccine to 100 μg and given it as a single dose by subcutaneous route animals presented a higher rate of protection than dogs immunized twice with the same vaccine, since 57% of dogs remained asymptomatic compared to 28% in the group that was immunized with two doses; through histopathological analyses, it was observed that the liver, spleen, and kidneys of the vaccinated dogs exhibited a normal histological morphology. Furthermore, vaccinated animals exhibited a microbicidal response, as evidenced by the elevated production of nitric oxide in lymph node cell cultures compared to non-immunized and infected animals [172]. In another field study, dogs were vaccinated with a single dose of Letifendi^®^ by subcutaneous injection (with a booster dose after one year), and no adverse reactions were observed. These dogs were housed in 19 kennels located in canine leishmaniasis endemic areas of France and Spain. Among the vaccinated dogs, 96.5% remained healthy, with no signs of infection; over the 780-day follow-up period, vaccinated dogs exhibited anti–Q protein IgG2 antibodies, with two significant increases observed after the initial vaccination and the booster dose.

Leish-111f^®^, also known as MML or LEISH-F1, is a vaccine produced with a chimeric *Leishmania* antigen, composed of three recombinant antigens: rTSA (*L. major*), LmSTI1 (*L. major*), and LeIF (*L. braziliensis*). The first study that described the efficacy was the MML vaccine to protect mice from the challenge with *L. major,* which was developed by Coler and collaborators (2002) [173]. It was demonstrated that vaccination of BALB/c mice with three subcutaneous doses containing 10 μg of MML did not induce a humoral immune response. In contrast, the association of MML with the adjuvant MPL-SE induced the production of IgG1 and IgG2a antibodies and stimulated IFN-γ secretion; therefore, this formulation was maintained for the challenge experiment. BALB/c and C57BL/6 mice were immunized following the same protocol and challenged, in the left hind footpad, three weeks after the last dose with metacyclic promastigotes of *L. major* or *L. amazonensis*, respectively [173]. Immunization prevented enlargement of the lesion in 80% of the *L. major*-infected mice and 60% of the *L. amazonensis*-infected mice. These results demonstrate that the vaccine had a prophylactic effect when formulated with MPL-SE [173]. However, studies combining Leish-111f^®^ with different adjuvants showed that MML + MPL-SE and MML + Adjuprime [90] were ineffective in protecting vaccinated dogs. The animals received three subcutaneous doses at 28-day intervals, and one year after the last dose, the vaccination regimen was repeated. The dogs were kept in open kennels located in an endemic area for human and canine visceral leishmaniasis in Italy. After two years of observation, 37 out of 39 dogs (95%) were infected with *L. infantum*, and MML failed to induce lymphocyte proliferative responses after the first vaccination schedule. These findings indicate that the antigen and adjuvant doses employed did not confer protection to the animals nor prevent disease progression [90]. It is important to highlight that field studies involve exposure to other pathogens that may increase immunological susceptibility to *Leishmania* infection, which may explain the discrepancies observed between the two studies, in addition to differences in the animal models used.

Another vaccine for canine leishmaniasis is Canileish^®^, produced in France by *Virbac*; this vaccine is also known as LiESP/QA-21, and consists of secreted-excreted proteins from *L. infantum* and QA-21 as adjuvant [168]. First, this antigen (LiESP) was formulated with adjuvant MDP and tested in dogs [174,175,176]. The animals were immunized with two subcutaneous doses. In two of the three studies, animals were challenged with *L. infantum* promastigote forms; in both studies, 100% of the vaccinated remained asymptomatic after the challenge, and the parasitic load was undetectable in the bone marrow. The third study was a field trial carried out in regions of southern France where leishmaniasis is highly prevalent [175]. The animals, comprising hunting, guard, and breeding dogs, were maintained in their owners’ households; after two years of follow-up, only 1 out of 165 vaccinated dogs (0.61%) was infected, whereas 12 out of 175 dogs in the placebo group (6.86%) were infected. Moreover, macrophages from vaccinated dogs produced higher levels of NO and IFN-γ compared to those from the placebo group. In a study conducted by Oliva and colleagues, dogs were vaccinated with three doses of the vaccine LiESP/QA-21 and kept in open kennels to allow exposure to sandflies [161]. They observed that the vaccine was well-tolerated by the animals, with the only adverse reaction associated with edema at the injection site. Among the vaccinated animals, 51% were undetectable for *Leishmania*, and 92% remained free of any disease symptoms.

The second generation of vaccines represents a substantial progress in the development of immunization against leishmaniasis. The isolation and characterization of parasite antigens such as LACK, GP63, histones, and HASPB, and others, provide insights into their immunogenic and antigenic potentials, enabling the selection or even the combination of antigenic factors within a single vaccine formulation. Although the methodology for attaining second-generation has led to the licensing of vaccines for canine leishmaniasis, their efficacy and safety remain unclear, and further studies are required to fill the existing knowledge gaps. Refinement in the selection of antigens and adjuvants, along with a deeper understanding of host-specific immunomodulation, is essential for the development of more effective vaccines against leishmaniasis. Table 5 summarizes the main findings about first-generation vaccines.

### 5.4. Third Generation Vaccines

Genetic vaccines comprise vaccination strategies performed with deoxyribonucleic acid (DNA vaccines) or messenger ribonucleic acid (mRNA) of the target protein of interest. This technology was initially developed in the 1990s and consisted of intramuscular injections with bacterial plasmid (DNA vaccine) encoding reporter genes, such as chloramphenicol acetyltransferase (CAT), luciferase, and beta-galactosidase, which, after inoculation in transfected cells, were able to express the target protein on the cell surface. In the case of mRNA vaccines, the initial concept was developed by Malone and collaborators, showing that mRNA could also be used to transfect a variety of eukaryotic cells that became able to express a bioactive protein, since these mRNA are given in correct carriers [188].

Basically, the idea of both types of biological products is that a given organism can produce a protein that is missing in biological systems. Although they share a similar technological concept, these vaccines work in different ways. DNA vaccines consist of a bacterial plasmid gene that contains a sequence that will encode an antigen under the control of a eukaryotic promoter, allowing gene expression in transfected cells. In general, plasmids contain an enhancer, a promoter, a transcription termination or polyadenylation signal sequence, and an origin of replication. To facilitate the growth and selection of transfected bacteria, an antibiotic marker is added to the plasmids.

The effectiveness of DNA vaccines depends on different factors, such as the antigen, type of vector, transfection method, number of injections, dose, and adjuvants. Despite several factors controlling the efficacy of a given vaccine, in general, formulations containing DNA vaccines have a similar action mechanism. After being transferred to the host cell (through conventional injection or a gene gun device, for example), the plasmid must enter the nucleus to be transcribed to mRNA. After this step, a bioactive protein will be translated into host cell ribosomes and subjected to post-translational modifications. Depending on the protein features, it can be secreted to the extracellular environment or processed by the major biochemical pathways of histocompatibility, activating the immune system [189].

Therefore, once produced, bioactive protein can be processed by the following pathways: (a) if protein is soluble into the host cell cytoplasm, it will be fragmented by the proteasome, and derived peptides will be presented into the cell surface by major histocompatibility complex I (MHCI), in consequence stimulating CD8^+^ T lymphocytes, (b) if produced protein is contained in specialized vacuoles of antigen-presenting cells (APC), it will be processed by MHC II machinery and derived peptides will be presented in cell surface in the context of MHC II molecules. In this case, the peptides will trigger CD4+ T cells [190]. Furthermore, DNA-encoded protein can also be processed as an exogenous antigen and presented by (c) MHCI during cross-presentation or (d) be captured by phagocytic cells and presented by MHCII molecules. All these mechanisms can occur together, triggering both cellular and humoral immune responses [191].

In contrast, an mRNA vaccine is not transported to the nucleus, but it is translated into protein directly by ribosomes in the cell cytoplasm. A classical mRNA vaccine contains a 5′ cap, made up of a 7-methylguanosine moiety followed by a triphosphate moiety close to the first nucleotide. This structure protects mRNA vaccines from host enzyme cleavage and regulates splicing. Other structural components of mRNA vaccines are the 5′ and 3′ untranslated regions (UTRs) that are positioned between the gene of interest and both the 5′ and 3′ ends. These regions are not translated into protein but regulate mRNA expression, by having an important role in recognizing mRNA by ribosomes; furthermore, such areas help the post-transcriptional modification of mRNA. Finally, the sequence of interest is added to the RNA vaccine, followed by the poly(A) tail, which is essential to protect RNA from degradation. To protect the RNA vaccine from degradation by innate mechanisms of immunity, vaccines are frequently encapsulated in vesicles containing common phospholipids [192].

Inside a target cell, the mRNA vaccine is going to be translated into proteins by the ribosomes. Depending on the signal peptide in the antigen, the protein will be processed by the MHC I machinery and the derived antigens presented to CD8^+^ cytotoxic T cells. In APC, the protein is directed to the MHC II compartment, and peptides will be presented on the surface of the APC to CD4+ T cells. Although mRNA vaccines have emerged as a promising immunoprophylactic strategy in the COVID era [102], this strategy was poorly explored in leishmaniasis, and thus, a great focus will be given to DNA vaccines in this section.

In leishmaniasis, the first gene to be assayed as a genetic vaccine was gp63. This gene was inserted into the plasmid pcDNAI, and the DNA vaccine produced was used to immunize BALB/c mice intramuscularly in the skeletal muscle of the thigh with 100 μg of vaccine. This study was a significant landmark for the development of DNA vaccines because it confirmed that immunized animals were able to express the bioactive protein in the cell membrane of muscle cells; in addition, this vaccine was immunogenic, considering the significant stimulation of the Th1 immune response. Importantly, this vaccine partially protected immunized animals and decreased cutaneous parasitism 100 times compared to the control [193].

Other studies also investigated the potential of gp63 in different formulations and vaccination strategies. The protective potential of the DNA vaccine pcDNA3.1-gp63 was compared with the recombinant protein as well as the heterologous prime-boost strategy, constituted with DNA vaccine and recombinant protein plus CpG adjuvant during the challenge performed with *L. major* in BALB/c mice. In general, it was observed that the heterologous prime-boost strategy, where animals were primed with two intramuscular immunization with pcDNA3.1-gp63 (50 μg/dose, two doses) plus CpG and boosted twice with recombinant gp63 plus CpG, presented the highest protective potential to BALB/c mice, furthermore, animals responded in a Th1 manner, considering the increase in IgG2a over IgG1 isotype levels. In contrast, other immunization strategies that used only pcDNA3.1-gp63 or recombinant protein in the presence of CpG adjuvant also exhibited protective properties; however, only the prime-boost method reduced hepatic and splenic parasitism in a long-term study, suggesting that, in addition to immunogenicity and protection, prime-boost immunization conferred immunological memory to immunized mice. This method potentiates a specific immune response because a DNA vaccine induces high-avidity T cells while recombinant protein boosts the immune system, allowing the proliferation of highly specific and active T cells [194]. The same vaccine also could restrain parasitism in BALB/c mice with visceral leishmaniasis caused by *L. donovani*; furthermore, this partial protection was associated with elevation in lymphoproliferative and cytotoxic responses, as well as IFN-γ and IL-2 cytokines [195]. Possibly, partial protection observed in such studies may be related to the maintenance of elevated levels of IL-4 and IL-10 [196]. In experiments using a gene gun, it was demonstrated that pcDNA3.1-gp63, given twice, was highly immunogenic and protective to BALB/c mice challenged with *L. mexicana* compared to the intramuscular route immunization; furthermore, this protection was associated with an increase in CD8^+^ T cell cytotoxicity [197].

In contrast, the genetic fragment of gp63, comprising the sequence between 138–360aa of this antigen, was cloned into the pVAX1 plasmid and given to BALB/c mice. Studies related to immunogenicity showed that this epitope-based vaccine increased the levels of both IgG1 and IgG2a isotypes, suggesting a mixed immune response. However, after challenge with *L. infantum,* a potent Th1 immune response was developed, with the participation of CD4^+^ and CD8^+^ T lymphocytes, as well as IFN-γ, IL12, and TNF-α. Additionally, a progressive decrease in inflammatory infiltrate and granulomas was observed along with a significant reduction in parasitism [198].

Another antigen administered as a third-generation vaccine is the *Leishmania* homologue of receptor for activated C kinase (LACK), also called p36. It is a 36-KDa protein highly conserved in the *Leishmania* genus and is expressed in both stages of the parasite. During the relationship between parasite and host, LACK plays an important role in the establishment of the Th2 immune response, ensuring the persistence of *Leishmania* in the host cell [199]. Therefore, considering that LACK is conserved in the *Leishmania* genus and plays a vital role in the establishment of infection, it could be an interesting target to develop a pan-vaccine for leishmaniasis.

In 1997, Gurunathan and colleagues cloned a truncated LACK sequence into the pcDNA3 plasmid and utilized it to immunize BALB/c mice in the hind footpad with 100 μg of vaccine by subcutaneous route with or without a eukaryotic expression vector carrying IL-12. In this case, it was observed that animals challenged with *L. major* promastigotes exhibited long-lasting protection, considering that the infection was controlled for 20 weeks after challenge. Furthermore, immunized mice controlled the development of lesions at sites distant to the place of immunization, suggesting that systemic protection as well as a significant development of immunological memory, which was associated with the CD8^+^ T lymphocyte expansion, was prompted after immunization with pcDNA3-LACK vaccine [200,201]. In contrast to this study, it was demonstrated that LACK cloned into the pcDNA3.1 vaccine expression vector does not protect BALB/c mice from the challenge with *L. donovani*, despite its immunogenic properties in animal models [202]. The same pattern was observed in mice immunized intramuscularly with a DNA vaccine produced with LACK cloned into pCI-neo and challenged with *L. chagasi* [203].

In experimental American cutaneous leishmaniasis, it was observed that the LACK gene cloned into pcI-neo administered intranasally twice (30 μg/dose), exhibited prophylactic efficacy in BALB/c mice challenged with the *L. amazonensis* parasite. This genetic vaccine significantly restricted parasite growth and, after 5 months of challenge, elevated levels of IFN-γ in both mucosa and draining lymph nodes, suggesting that this DNA vaccine elicited long-lasting immunity in immunized animals [204]. This same formulation and dose also administered by the mucosal route protected BALB/c mice from challenge with *L. infantum*, which developed significant Th1 and Th2 immune responses, along with a reduction in IL-10 levels, which is essential to ensure protective immunity in visceral leishmaniasis [205]. Using hamsters, which is an animal model that resembles the natural infection of VL more significantly, it was proven that this experimental vaccine also protected animals challenged with *L. infantum* [206]. More recently, it was demonstrated that this same vaccine formulated in crosslinked chitosan microparticles was safe after administration to mice intranasally, and protection in models of visceral and cutaneous leishmaniasis was potentized compared to the free vaccine [207,208], suggesting that nanocarriers can be a significant platform for vaccine delivery.

In consideration of the high immunogenicity and protective properties of this vaccine, a heterologous prime-boost strategy was developed, in which the LACK gene was inserted into the pCI-neo plasmid and into an attenuated modified virus Ankara (MVA). LACK-pCI-neo was injected intradermally, and two weeks later, recombinant Ankara virus expressing LACK was injected intraperitoneally. This strategy increased the levels of LACK-specific IFN-g-secreting cells as well as IFN-γ and TNF-α-producing CD4^+^ and CD8^+^ T lymphocytes. Despite the Th1 immune response elicited by this strategy, elevated IL-4 levels were detected, suggesting that this vaccine and immunization strategy is highly immunogenic but causes a mixed immune response in the host, which possibly accounted for the partial protection observed after challenge with *L. major*, however, this protection was maintained during 17 weeks post challenge, suggesting that the vaccine elicited immunological memory [209]. In the hamster model of VL caused by *L. infantum*, it was demonstrated that the same strategy triggers protection [148].

A similar pattern was observed in canine visceral leishmaniasis; in this case, the beagle dogs were first immunized with 100 μg of the plasmid vector pORT-LACK and 15 days later with 10^8^ pfu of MVA-LACK by the subcutaneous route. This immunization strategy induced a reduction in clinical symptoms as well as hepatic parasitism; however, did not cause a sterile cure in animals [210]. LACK was also inserted into the non-replicative antibiotic-resistant-free pPAL plasmid, and then used to immunize beagle dogs. pPAL-LACK vaccine was immunogenic and induced a significant increase in a Th1 immune response; however, animals challenged with *L. infantum* exhibited only partial protection after immunization, possibly an imbalance between IFN-γ and IL-10 causes this partial protection [211].

Another well-studied target for the development of genetic vaccines is leishmanial ribosomal proteins. In general, the ribosome translates genetic information into proteins that have different biological roles in parasites. The ribosome structure consists of four ribosomal RNA molecules as well as different associated ribosomal proteins that form large and small ribosomal subunits [212]. In the context of immunization, the development of vaccines containing ribosomal genes or even proteins [213] has been considered a significant strategy, because many of these antigens are conserved in the *Leishmania* genus [214]; therefore, a multivalent vaccine can be produced.

The first ribosomal protein studied as a genetic vaccine was the acidic ribosomal protein P0 of *L. infantum*, where the genetic sequence was cloned into pcDNA3 (pcDNA3-LiP0). This genetic vaccine was inoculated intramuscularly in the quadriceps (50 μg per leg) of BALB/c mice twice. Fourteen days after the last dose, the animals were challenged with *L. major* promastigotes in the footpad. Animals immunized with this genetic vaccine exhibited a delay in the progression of the cutaneous lesion and a reduction in parasitism. Furthermore, the protective mechanism of this vaccine is related to the priming of CD4^+^ and CD8^+^ T lymphocytes as well as high production of IFN-γ [215]. This same vaccine was assayed in visceral experimental leishmaniasis caused by *L. infantum* in golden hamsters; in this regard, a similar protective pattern was observed, where animals that received three doses of pcDNA3-LiP0 intramuscularly (100 μg/dose) exhibited a significant reduction in hepatic and splenic parasitism along with upregulation of a Th1 immune response, furthermore, the liver and spleen presented fewer histopathological changes compared to the infected control [216].

Another gene assayed as a DNA vaccine was the ribosomal P1 gene. The open reading frame of the *L. donovani* ribosomal P1 gene was cloned into the pVAX1 plasmid (pVAX1-P1). The hamsters were immunized intramuscularly once or twice and challenged with *L. donovani* promastigotes intracardially two weeks after the last dose. Compared to unvaccinated infected animals, vaccinated animals with pVAX1-P1 were observed to have reduced liver and spleen weight that was correlated with a low parasitism and a high ratio between IFN-γ/IL-4 and IL-12/IL-10, as well as proliferation of splenocytes [72].

**Figure 7:**
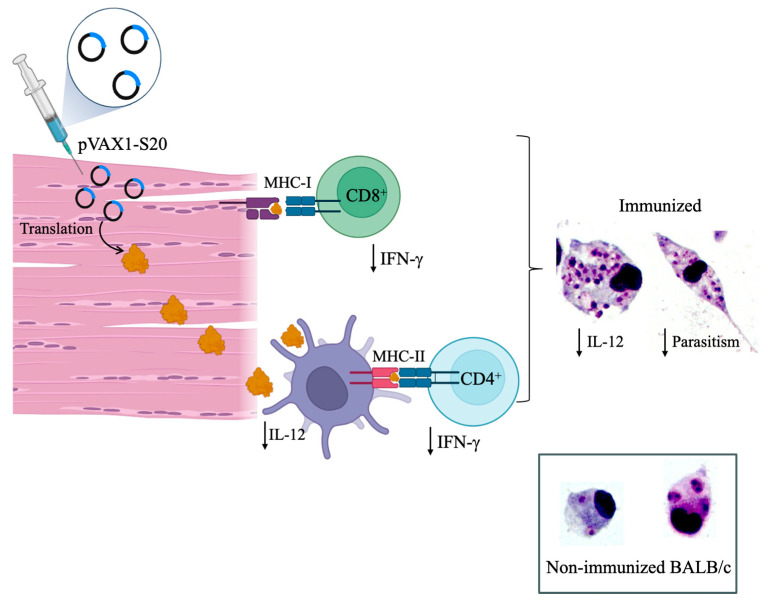
Immunogenicity of the S20 ribosomal antigen formulated as a DNA vaccine. BALB/c mice were immunized with the third-generation vaccine produced with the S20 ribosomal gene inserted in the pVAX-1 plasmid. BALB/c mice were immunized three times with 100 mg/animal/animal intramuscularly. Fifteen days after the last injection, the T cell and macrophage responses were analyzed. In this case, pVAX1-S20 reduced the Th1 and CD8^+^ T activities, allowing the multiplication of amastigote forms in macrophages that produced low amounts of IL-12 cytokines.

More recently, our research group studied the immunogenicity of the S20 ribosomal protein, whose open genetic reading frame was inserted into the pVAX1 vector. This vaccine was administered in BALB/c mice intramuscularly, three times in a 2-week interval. It was observed that BALB/c mice produced antibodies that specifically recognized a protein with 14 kDa, corresponding to the molecular mass of S20 ribosomal protein, suggesting that immunized animals expressed a bioactive protein. Immunologically, increased frequencies of T lymphocytes were detected, which was correlated with a significant enhancement of TNF-α-producing CD8^+^ T lymphocytes, suggesting that this genetic vaccine has stimulated cytotoxic T lymphocytes. Despite that, it was observed that IFNγ-producing CD4^+^ and CD8^+^ T cells decreased by six and seven times in comparison to the control, suggesting that this antigen has immunosuppressive potential (Passero et al., data not published). Furthermore, peritoneal macrophages from the immunized group were more permissive to *L.* (*L.*) *amazonensis* infection than controls, confirming that this antigen has suppressive potential (Figure 7).

Another class of antigens investigated as genetic vaccines is related to the antioxidant machinery of *Leishmania* sp. This machinery is a set of antioxidant enzymes responsible to detoxify reactive oxygen and nitrogen species, such as hydrogen peroxide, hydroxyl radicals, superoxide anion, nitric oxide, among others, which are capable of damaging lipids, proteins, and nucleic acids, leading parasites to death; in general, these molecules are produced during an immune response or even as a product of drug metabolism [217].

In this regard, it has been well established that the antioxidant machinery of *Leishmania*, as well as other tripanosomatids, is complex and involves the participation of different enzymes located in several compartments. Iron superoxide dismutase (SOD) detoxifies the superoxide radical into oxygen and hydrogen peroxide, which in turn is detoxified by other enzymes with peroxidase activity, such as tryparedoxin peroxidase (TR). In the context of genetic vaccination, it was demonstrated that TR inserted in the plasmid pcDNA3 (pcDNA3-TR) and in a modified vaccinia virus Ankara (MVA-TR) was immunogenic to BALB/c mice. In this case, the animals were subcutaneously immunized in the rump with 100 μg of pcDNA3-TR at a 3-week interval. Five weeks later, the animals were boosted by the intravenous route with 1 × 10^6^ PFU of MVA-TR. In general, immunization was observed to cause a significant increase in IFN-γ levels that was correlated with partial protection after challenge with *L. major*. Additionally, it was observed that this strategy induced long-lasting protection, considering that 16 weeks after boost, BALB/c mice still partially controlled lesion development, which was associated with increased IFN-γ and IL-4 production [218].

The same prime-boost strategy using the DNA vaccine and recombinant MVA was employed in the infection caused by *L.* (*V.*) *panamensis*. In this case, TR was cloned into pVAX-1. This strategy showed that this vaccine increased the frequencies of IFN-γ-producing CD4^+^ and CD8^+^ T cells, which accounted for a reduced size of the cutaneous lesion in challenge mice as well as low parasite loads in the skin and lymph nodes. Furthermore, this vaccine seems to stimulate CD8^+^ T lymphocyte clones, considering that their depletion led to a rapid progression of the lesion; in contrast, the depletion of CD4^+^ T lymphocytes did not alter the protective effect of vaccination [219].

Due to the immunogenicity and protective potential of the TR vaccination, its immunogenicity was evaluated in outbred dogs. In this case, it was demonstrated that the prime-boost strategy using DNA vectors and MVA was safe and immunogenic for dogs, considering that immunized animals produced higher levels of IFN-γ and IgG2a, pointing to a specific and significant Th1 immune response, responsible for activating macrophages [218].

The SOD enzyme is an important molecule responsible for the conversion of superoxide radicals into oxygen and hydrogen peroxide. SOD requires a metal cofactor, such as iron, in the active site to become active [220]. Iron-superoxide dismutase is classified according to the cytoplasmic compartment in which it accumulates. SOD-A and SOD-C can be found in the mitochondria, while SOD-B1 and SOD-B2 can be found in the glycosomes [221,222,223]. This enzyme is a promising antigen to be formulated as a leishmania vaccine because it is absent in humans; thus, severe adverse reactions or even autoimmune reactions are not expected during the vaccination process. Despite work dealing with second-generation vaccines that employ SOD as a prototype vaccine, few works have explored the ability of this class of antigens to be used as a third-generation vaccine.

In this regard, it was demonstrated that a prime-boost strategy performed with a DNA vaccine and recombinant SOD protein was performed in BALB/c mice. Animals were injected intramuscularly with two doses of pcDNA (100 μg plus 25 μg of CpG ODN 1826/animal) containing *L. donovani*. After 15 days, the animals received one dose of recombinant protein (12.5 μg plus 25 μg of CpG ODN 1826/animal) subcutaneously. It was observed that pcDNA-SOD was immunogenic to mice, inducing a high level of anti-SOD IgG, especially IFN-γ and IgG2a in pcDNA-SOD and pcDNA-GMCSF-SOD groups. Furthermore, enhancement of TNF-α- and IL-2-producing CD4^+^ T lymphocytes along with T cytotoxic 1 and 2 cell subsets was observed in both groups, culminating in a partial protection after challenge with *L. major*, as judged by the size of lesions of animals [224]. Although IL-10 levels have been reduced during infection with *L. major* in immunized animals, the amount still detected should be enough to inhibit the Th1 immune response, allowing parasites to escape from an effective immune response and survive in the host.

Another study showed that the SOD gene inserted in the pVAX1 plasmid (pVAX1-SOD) was also immunogenic when injected three times (100 μg/animal) intramuscularly in BALB/c mice. In this work, animals that received three doses of pVAX1-SOD exhibited high frequencies of IFN-γ-producing CD4^+^ T cells as well as IFN-γ and IL-4-producing CD8^+^ T lymphocytes, along with high levels of IgG2a isotype, indicating a protective immune response. After challenge, *L. amazonensis* parasites abrogated the expansion of IFN-γ-producing CD4^+^ T cells, observed in immunogenicity studies, but also led to an expansion of IL4-producing CD4^+^ T lymphocytes. In contrast, a significant expansion of IFN-γ-producing CD8^+^ T cells was observed, suggesting that partial protection was achieved by the cytotoxic subset of T cells [225]. Another antioxidant enzyme, the thiol-specific antioxidant, formulated as a DNA vaccine, also protected partially challenged mice, possibly due to a significant increase in IL-4 cytokine levels [226,227].

Although antioxidant enzymes seem to be excellent alternatives for producing third-generation vaccines, considering their immunogenicities and the absence of such genes in humans to minimize toxic events, the parasites were able to evade the immune response triggered by experimental immunizations. Possibly, the antioxidant enzyme and other important virulence factors present in viable parasites inhibited the inflammatory environment triggered by just one specific antigen.

From the perspective of DNA vaccines, different studies also investigated the immunogenicity and protective potential of bivalent or multivalent DNA vaccines in preclinical studies using mice, hamsters, or dogs as hosts of cutaneous or visceral species of *Leishmania*. Examples of such vaccines are TSA and LmSTI1—*L. major* homolog to the eukaryotic stress-inducible protein [228]; PSA-2, peroxidoxin/TSA, STI1, ARP-1 along with a cocktail of histones [229]; LACK-TSA [227]; KMP11, TSA, CPA, CPB and P74 sequences [230]; and KMPII, TRYP, LACK and GP63 [231]. This strategy is interesting because it is possible to put together different genes or genetic fragments, theoretically assuring protection against different species of *Leishmania* in hosts with different HLA alleles, therefore, a DNA vaccine containing multiple genes or genic segments would induce a protective immunity in a broad range of individuals [232,233], suggesting that this is a significant strategy to develop a protective vaccine to combat all forms of leishmaniasis. Despite the benefits of producing such DNA vaccines, studies have already demonstrated that only partial protection was achieved after challenge with viable parasites or, in the same cases, failed to protect dogs from an experimental challenge [218]. Table 6 summarizes the main findings on third-generation vaccines.

## 6. Conclusions

This revision covered different aspects of leishmaniasis, summarizing studies related to the relationship between parasites and hosts, immunology, clinical manifestations, experimental models, adjuvants, and vaccines. By describing these areas of knowledge, it was possible to highlight many elegant works developed toward understanding this complex infectious disease. Despite these advances, in some cases, it is not possible to predict the type of disease that a parasite species will cause during a natural infection in humans. This is, in fact, an association of factors, such as the infecting parasite species (and strain), vector species, the physiological status of the vertebrate host, and genetics. Together, these factors determine the course of the disease and the expression of its clinical form.

At the same time, such elements can also influence the efficacy of vaccines in biological systems. It is also important to mention that *Leishmania* genus exhibits a great biodiversity of species, which is associated with molecular diversity; this means that phenotypic differences exist between immunogenic proteins, impacting the immunogenicity and protective potential of vaccines. Furthermore, the existence of strains with atypical behavior, such as *L. infantum chagasi* causing non-ulcerated cutaneous leishmaniasis in Central America, negatively impacts the development of a universal vaccine.

Regarding immunizations, animals are initially injected with a specific antigen and can respond with a significant protective immune response. However, after a challenge with an artificially increased number of parasites, the immune system must struggle with a multitude of parasitic antigens, making it hard to respond specifically, rapidly, and protectively to a single antigen. In this review, all described immunogens conferred partial or no protection. Could these responses be caused by an excessive amount of parasitic antigen in these biological systems? Although these experimental models are important and have contributed to solving immunological puzzles related to leishmaniasis, it would be beneficial to develop new models that better resemble human disease.

Concerning *Leishmania* vaccines, this review highlights that conserved subunit vaccines are significant, as some important examples of commercialized vaccines originate from the second generation of vaccines. For instance, immunogens employing saponin as an adjuvant are notable at protecting dogs from the infection. In fact, studies on malaria vaccines also indicate that second-generation vaccines can be safely administered to humans and are protective, as evidenced by the development of two important immunogens approved by the WHO to block malaria transmission in humans. Finally, further studies on *Leishmania* vaccine should align the biology of natural transmission with the principle of a strong and long-lasting cellular immunity, which certainly would block the transmission of *Leishmania* parasites between invertebrate and vertebrate hosts.

## Figures and Tables

**Table 1:** Summary of experimental models used in visceral leishmaniasis.

Animal Model	Causative Agent	Key Features of VL Infection	Relevance to Human VL	Limitations	References
Dog (*Canis familiaris*)	*L.* (*L.*) *infantum*	Natural domestic reservoir Infection mimics human VL clinical progression Symptoms: weight loss, anemia, lymphadenopathy, fever Skin lesions present (absent in humans) Parasites in spleen and skin even in asymptomatic dogs	Similar disease progression Important for epidemiology and experimental models Useful for vaccine and drug testing	High maintenance cost Ethical concerns Limited animal numbers per study	[23,24,25,26,27,30,35]
Mouse (*mus musculus*)	*L.* (*L.*) *donovani* and *L.* (*L.*) *infantum*	Subclinical and limited infection Effective cellular immune response Chronic fever, hepatosplenomegaly, cachexia absent Useful for studying protective immunity and genetic factors	Model for self-controlled or oligosymptomatic human cases Widely used due to availability of immunological tools	Does not fully reproduce human VL pathology	[36,37]
Golden hamster (*mesocricetus auratus*)	*L.* (*L.*) *donovani*	Highly susceptible to *Leishmania* Develops cachexia, hepatosplenomegaly, pancytopenia, hypergammaglobulinemia Granulomas in liver, progressive spleen infection Failure to produce NO despite Th1 cytokine response - Ascites and renal complications rare in humans	Reproduces many human VL clinical and immunopathological features	Limited immunological reagents Some pathological features not typical of humans	[28,29,38,39]
Rhesus macaque (*Macaca mulatta*)	*L.* (*L.*) *infantum*	Systemic disease similar to human VL Symptoms: fever, diarrhea, weight loss, anemia, hypergammaglobulinemia, lymphadenopathy, hepatosplenomegaly Hepatic granuloma formation with epithelioid and Langhans giant cells Useful for vaccine and drug evaluation	High phylogenetic similarity Similar immune responses Mimics human infection	High cost and ethical issues Complex maintenance Some differences in disease control	[34]
*Cebus apella* monkey	*L.* (*L.*) *infantum chagasi*	Transient infection Effective cellular immune response controls parasite spread	Not recommended to mimic natural infection but useful to study resistance mechanisms	Does not adequately mimic human natural infection	[40]

VL—visceral leishmaniasis.

**Table 2:** Summary of experimental models used in cutaneous leishmaniasis.

Experimental Model	Causative Agent	Human Clinical Form(S)	Model Characteristics	Relevance to Human Disease	Limitations	References
MOUSE (*MUS MUSCULUS*, BALB/C)	*L*. (*L*.) *amazonensis*	LCL MCL (rare) DCL	Progressive ulcerative lesions cutaneous metastasis Th2-type immune response associated with susceptibility	Mimics progressive disease in some human cases Useful for immunological studies	Limited chronic disease features	[46]
MOUSE (*MUS MUSCULUS*, C57BL/10, C57BL/6, DBA/2)			Chronic disease or subclinical infection depending on strain Eosinophil and mast cell infiltration. Th1 or Th2 immune responses	Mimics spectrum of human disease Allows study of genetic and immune response variability	Strain-dependent variability; some immune responses differ from humans	[47,48]
*RHESUS* MONKEYS (*MACACA MULATTA*)			Progressive ulcerative lesions Lesions display mononuclear cell infiltrates composed of macrophages, lymphocytes, and plasma cells, along with tuberculoid-type granuloma formation There is a shift in circulating T cell subpopulations, initially a predominance of CD4^+^ T cells, followed by an increase in CD8^+^ T cells as the infection progresses	Mimics progressive of the cutaneous leishmaniasis in humans Useful for immunological studies Useful to evaluate the efficacy of new vaccines	High cost and ethical issues Complex maintenance Limited availability and sample sizes Longer experimental timelines	[49]
MOUSE (*MUS MUSCULUS*, BALB/C)	*L*. (*V*.) *braziliensis*	LCL MCL	Transient or ulcerated lesions depending on inoculation site (hind footpad or ear)	Some infection features resemble human disease including lesion ulceration and parasite persistence	Resistance in many mouse strains; difficulty obtaining infectious stages	[42,51]
*SAPAJUS APELLA* MONKEYS			Parasite persistence during infection Cutaneous lesions present	Mimics human infection dynamics well. Useful for immunopathology and vaccine studies	High cost and ethical issues Complex maintenance Limited availability and sample sizes Longer experimental timelines	[50]
MOUSE (*MUS MUSCULUS*, BALB/C)	*L*. (*V*.) *panamensis*	LCL Rare mucosal involvement	Lower virulence observed in rodents	Limited data Less commonly used models	Low virulence limits study of full disease spectrum	[43]
MOUSE (*MUS MUSCULUS*, BALB/C)	*L*. (*V.*) *shawi*	LCL	Higher susceptibility with footpad swelling Severe inflammation Th1 response	Represents natural infection in humans Useful for drug and vaccine development	Species-specific response; may not represent all human cases	[12]

LCL: Localized cutaneous leishmaniasis; MCL: Mucocutaneous leishmaniasis; DCL: Diffuse cutaneous leishmaniasis.

**Table 3:** Leishmanization against leishmaniasis over the past 20 years.

Name	Vaccine Parasite	Adjuvant	Host	Route	Doses	Challenge	Protection	Reference
Ld	*L. donovani*		BALB/c	Subcutaneous	Single dose	*L. donovani*	<50% less liver parasite burden	[120]
Li	*L. infantum*		Dog	Subcutaneous	Single dose	*L. infantum*	100% without *Leishmania* DNA; 97.8% without clinical signs	[115]
Li HSP70-II^−/−^	*L. infantum*		BALB/c	Subcutaneous	Single dose	*L. major*	90% less spleen parasite burden	[117]
Lm	*L. major*	CpG	C57BL/6	Intradermal	Single dose		100% without lesion	[110]
Lm	*L. major*		BALB/c	Subcutaneous	Single dose	*L. major*	50% less skin parasite burden	[122]
Lm Cen^−/−^	*L. major*		Hamster	Intradermal	Single dose	*L. donovani*	90% less liver and spleen parasite burden	[118]
Lm tkcd^+/+^	*L. major*		C57BL/6	Subcutaneous	Single dose	*L. major*	90% spleen parasite burden; 100% without lesion	[116]
Lt	*L. tarentolae*		BALB/c	Intraperitoneal	Single dose	*L. donovani*	80% spleen and liver parasite burden	[113]
Lt	*L. tarentolae*	CpG	BALB/c	Intraperitoneal	Single dose	*L. major*	87% spleen parasite burden	[114]
Lt	*L. tarentolae*		BALB/c	Intraperitoneal	Single dose	*L. infantum*	90% spleen and liver parasite burden	[112]

**Table 4:** First-generation vaccines against leishmaniasis over the past 20 years.

Name	Vaccine Parasite	Adjuvant	Host	Route	Doses	Challenge	Protection	Reference
KBMA Li	*L. infantum*		BALB/c	Subcutaneous	3 doses	*L. infantum*	30% less liver parasite burden	[138]
La	*L. amazonensis*		C57BL/6	Intranasal	2 doses	*L. amazonensis*	50% less lesion size and parasite burden	[139]
La	*L. amazonensis*	ADDAVAX^®^	C57BL/6	Intranasal	2 doses	*L. amazonensis*	Ineffective	[139]
La	*L. amazonensis*	Saponin	BALB/c	Subcutaneous	3 doses	*L. chagasi*	25% less liver parasite burden; 50% less spleen parasite burden	[128]
La-phVac and La-mtVac	*L. amazonensis*		C57BL/10	Subcutaneous	2 doses	*L. amazonensis*	50% without lesion	[123]
Lb	*L. braziliensis*	Saponin	Dog	Subcutaneous	3 doses		Indeterminate	[129]
Lb	*L. braziliensis*	Saponin	BALB/c	Subcutaneous	3 doses	*L. chagasi*	25% less liver parasite burden; 50% less spleen parasite burden	[128]
Lb-Sal	*L. braziliensis*	Saponin	Dog	Subcutaneous	3 doses		Indeterminate	[130]
Lc	*L. chagasi*	Saponin	BALB/c	Subcutaneous	3 doses	*L. chagasi*	90% less liver and spleen parasite burden	[140]
Ld	*L. donovani*		BALB/c	Intraperitoneal	3 doses	*L. donovani*	15% less liver parasite burden; 24% less spleen parasite burden	[141]
Ld	*L. donovani*	BCG	BALB/c	Intraperitoneal	3 doses	*L. donovani*	30% less liver parasite burden; 40% less spleen parasite burden	[141]
Ld	*L. donovani*	MPL/TDM	BALB/c	Intraperitoneal	3 doses	*L. donovani*	30% less liver parasite burden; 40% less spleen parasite burden	[141]
Ld-Lip	*L. donovani*	Cationic liposomes		Intraperitoneal	3 doses	*L. donovani*	80% less liver parasite burden; 60% less spleen parasite burden	[141]
Leishvacin^®^	*L. amazonensis*		Human	Intramuscular	3 doses	*L. panamensis*	Indeterminate	[132]
Li	*L. infantum*	BCG	Dog	Subcutaneous	3 doses		Indeterminate	[142]
Li	*L. infantum*	AdjuPrimeTM	Dog	Subcutaneous	3 doses		Indeterminate	[142]
Li	*L. infantum*	MPL/TDM/CWS	Dog	Subcutaneous	3 doses		Indeterminate	[142]
Lm	*L.major*	BCG	BALB/c	Subcutaneous	3 doses	*L. major*	50% less lesion size; 90% less skin parasite burden	[124]
Lm	*L. major*	Alum	BALB/c	Subcutaneous	3 doses	*L. major*	50% less lesion size; 70% less skin parasite burden	[124]
Lm	*L.major*	Montanide ISA 720	BALB/c	Subcutaneous	3 doses	*L. major*	80% less lesion size; 90% less skin parasite burden	[124]
Lm	*L.major*	BCG	Human	Intradermal	Single dose		Indeterminate	[137]
Lm	*L. major*	CpG	BALB/c	Subcutaneous	3 doses	*L. major*	50% less skin parasite burden	[122]
Lm(FKP)	*L. major*		BALB/c	Subcutaneous	3 doses	*L. major*	50% less lesion size; 30% less skin parasite burden	[124]
sTLAVac	*L. amazonensis*		C57BL/6	Subcutaneous	2 doses	*L. amazonensis*	Indeterminate	[125]
LsAmaAg	*L. shawi*		BALB/c	Subcutaneous	2 doses	*L. shawi*	Ineffective	[134]
LsProAg	*L. shawi*		BALB/c	Subcutaneous	2 doses	*L. shawi*	60% less lesion size; 70% less skin parasite burden	[134]
WPV	*L. amazonensis* and *L. braziliensis*	BCG	Dog	Subcutaneous	3 doses		Indeterminate	[131]

**Table 5:** Second-generation vaccines against leishmaniasis over the past 20 years.

Name	Vaccine Parasite	Adjuvant	Host	Route	Doses	Challenge	Protection	Reference
LsPass1	*L. shawi*		BALB/c	Subcutaneous	2 doses	*L. shawi*	Ineffective	[157]
LsPass2	*L. shawi*		BALB/c	Subcutaneous	2 doses	*L. shawi*	50% less lesion size; 70% less skin parasite burden	[157]
LsPass3	*L. shawi*		BALB/c	Subcutaneous	2 doses	*L. shawi*	75% less lesion size; 90% less skin parasite burden	[157]
LiRic1	*L. infantum*		BALB/c	Intraperitoneal	4 doses	*L. infantum*	50% less spleen parasite burden	[156]
LiRic2	*L. infantum*		BALB/c	Intraperitoneal	4 doses	*L. infantum*	66% less spleen parasite burden	[156]
CaniLeish^®^/ESP	*L. infantum*	QA-21	Dog	Subcutaneous	3 doses		92.7% healthy	[161]
ChimeraT	*Leishmania*	Saponin	BALB/c	Subcutaneous	3 doses	*L. infantum*	80% less liver and spleen parasite burden; 90% less bone marrow and lymph node parasite burden	[155]
His6/LACK	*L. braziliensis*	CpG+Alumen	BALB/c	Intraperitoneal	3 doses	*L. braziliensis*	Ineffective	[177]
His6/LbSTI	*L. braziliensis*	CpG+Alumen	BALB/c	Intraperitoneal	3 doses	*L. braziliensis*	Ineffective	[177]
His6/LeIF	*L. braziliensis*	CpG+Alumen	BALB/c	Intraperitoneal	3 doses	*L. braziliensis*	Ineffective	[177]
His6/TSA,	*L. braziliensis*	CpG+Alumen	BALB/c	Intraperitoneal	3 doses	*L. braziliensis*	Ineffective	[177]
LaPSA-38S	*L. amazonensis*		In vitro				Indeterminate	[178]
LaSP	*L. amazonensis*	Saponin	BALB/c	Intramuscular	3 doses	*L. amazonensis*	Ineffective	[179]
Ld31	*L. donovani*	Cationic liposomes	BALB/c	Intraperitoneal	3 doses	*L. donovani*	70% less liver and spleen parasite burden	[152]
Ld51	*L. donovani*	Cationic liposomes	BALB/c	Intraperitoneal	3 doses	*L. donovani*	70% less liver and spleen parasite burden	[152]
Ld72	*L. donovani*	Cationic liposomes	BALB/c	Intraperitoneal	3 doses	*L. donovani*	60% less liver and spleen parasite burden	[152]
Ld78	*L. donovani*	Cationic liposomes	BALB/c		3 doses	*L. donovani*	97% less liver parasite burden	[152]
Ld79	*L. donovani*	MPL	BALB/c		3 doses	*L. donovani*	96% less liver parasite burden	[153]
Ld91	*L. donovani*	Cationic liposomes	BALB/c	Intraperitoneal	3 doses	*L. donovani*	40% less liver and spleen parasite burden	[152]
LdGP63	*L. donovani*	Cationic liposomes	BALB/c	Intraperitoneal	3 doses	*L. donovani*	80% less liver and spleen parasite burden	[147]
LdPDI	*L. donovani*		Hamster	Intramuscular	3 doses	*L. donovani*	90% less spleen, liver and bone marrow parasite burden	[180]
LdrA2	*L. donovani*	Saponin	Dog	Subcutaneous	3 doses		43% no parasite burden detected; 72% asymptomatic	[178]
LdTPI	*L. donovani*		Hamster	Intramuscular	3 doses	*L. donovani*	~90% less spleen and liver parasite burden	[181]
Leish-111f(MML) or LEISH-F1	*Leishmania*	CpG	C57BL/6	Subcutaneous	3 doses	*L. major*	83% less lesion size; 45% less skin parasite burden	[158]
Leish-111f(MML) or LEISH-F1	*Leishmania*	MPL-SE	Dog	Intradermal	3 doses	*L. infantum*	29% healthy	[146]
Leish-111f(MML) or LEISH-F1	*Leishmania*	MPL-SE	Dog	Subcutaneous	3 doses	*L. infantum*	Ineffective	[90]
Leish-111f(MML) or LEISH-F1	*Leishmania*	Adjuprime	Dog	Subcutaneous	3 doses	*L. infantum*	Ineffective	[90]
Leish-111f(MML) or LEISH-F1	*Leishmania*	MPL-SE	Human	Subcutaneous	3 doses		Indeterminate	[182]
Leish-111f(MML) or LEISH-F1	*Leishmania*	MPL-SE	Human	Subcutaneous	3 doses		Indeterminate	[183]
Leishmune^®^	*L. donovani*		Dog	Subcutaneous	3 doses		100% asymptomatic	[184]
Leishmune^®^/FML	*L. donovani*	Saponin	Dog	Subcutaneous	3 doses		89% healthy	[166]
Leishmune^®^/FML	*L. donovani*	Saponin	Dog	Subcutaneous	3 doses		100% asymptomatic; 100% without *Leishmania* DNA	[162]
Leishmune^®^/FML	*L. donovani*	Saponin	Dog	Subcutaneous	3 doses		Indeterminate	[165]
LeishTec^®^/rA2	*Leishmania*	Saponin	Dog	Subcutaneous	3 doses		92% healthy	[166]
LeishTec^®^/rA2	*Leishmania*	Saponin	Dog	Subcutaneous	3 doses		69% healthy	[160]
LeishTec^®^/rA2	*Leishmania*	Saponin	Dog	Subcutaneous	3 doses		63.7% healthy	[167]
LetiFend^®^/Q protein	*L. infantum*		Dog	Subcutaneous	Single dose		95.3% healthy	[159]
Q protein	*Leishmania*		Dog	Subcutaneous	Single dose		57% asymptomatic	[172]
Q protein	*Leishmania*		Dog	Subcutaneous	2 doses		28% asymptomatic	[172]
Li ESAp-MDP	*L. infantum*	MDP	Dog	Subcutaneous	2 doses	*L. infantum*	100% less bone marrow parasite burden	[176]
Li ESAp-MDP	*L. infantum*	MDP	Dog	Subcutaneous	2 doses	*L. infantum*	100% less bone marrow parasite burden	[174]
Li ESAp-MDP	*L. infantum*	MDP	Dog	Subcutaneous	2 doses		100% less bone marrow parasite burden; 91% without Leishmania DNA	[175]
LiH1	*L. infantum*	Montanide ISA 720	Dog	Intradermal	3 doses	*L. infantum*	62,5% healthy	[146]
LiH1-HASPB1	*L. infantum*	Montanide ISA 720	Dog	Intradermal	3 doses	*L. infantum*	50% healthy	[146]
LiH1-SMT-Hy-SAP	*L. infantum*		BALB/c	Subcutaneous	2 doses	*L. infantum*	80% less spleen parasite burden	[154]
LiH1-SMT-Hy-SAP	*L. infantum*	Freund	BALB/c	Subcutaneous	2 doses	*L. infantum*	80% less spleen parasite burden	[154]
LiHASPB1	*L. infantum*	Montanide ISA 720	Dog	Intradermal	3 doses	*L. infantum*	50% healthy	[146]
LiPHB	*L. infantum*	Saponin	BALB/c	Subcutaneous	3 doses	*L. infantum*	60% less liver, spleen and lymph node parasite burden; 75% less bone marrow parasite burden	[185]
LmLACK	*L. major*		BALB/c	Subcutaneous	2 doses	*L. major*	Ineffective	[144]
LmTRYP	*L. major*		BALB/c	Subcutaneous	2 doses	*L. major*	20% less lesion size	[144]
LPG3-IFA	*L. chagasi*	Saponin	BALB/c	Subcutaneous	3 doses	*L. chagasi*	96% less liver and spleen parasite burden	[186]
LPG3-Sap	*L. chagasi*	Saponin	BALB/c	Subcutaneous	3 doses	*L. chagasi*	98% less liver and spleen parasite burden	[186]
NGP5B/αGal		CpG	C57BL/6	Subcutaneous	4 doses	*L. major*	72% less skin parasite burden	[187]
rPHB	*L. infantum*	Saponin	BALB/c	Subcutaneous	3 doses	*L. infantum*	60% less liver parasite burden; 66% less spleen parasite burden and lymph node; 75% less parasites in the bone marrow;	[185]
LdGP63	*L. donovani*		BALB/c	Subcutaneous	2 doses	*L. donovani*	85% less spleen parasite burden; 68% less liver parasite burden	[150]
LdHsp70	*L. donovani*		BALB/c	Subcutaneous	2 doses	*L. donovani*	50% less spleen parasite burden; 40% less liver parasite burden	[150]
LdGP63-Hsp70	*L. donovani*		BALB/c	Subcutaneous	2 doses	*L. donovani*	90% less spleen parasite burden; 94% less liver parasite burden	[150]
LdGP63-Hsp70	*L. donovani*	MPL-A	BALB/c	Subcutaneous	2 doses	*L. donovani*	94% less spleen parasite burden; 97% less liver parasite burden	[151]
LdGP63-Hsp70	*L. donovani*	ALD	BALB/c	Subcutaneous	2 doses	*L. donovani*	92% less spleen parasite burden; 93% less liver parasite burden	[151]
LdLACK	*L. donovani*		BALB/c	Intraperitoneal	3 doses	*L. donovani*	Ineffective	[145]
LdLACK	*L. donovani*	Liposome	BALB/c	Intraperitoneal	3 doses	*L. donovani*	Ineffective	[145]
LdGP63	*L. donovani*	Liposome	BALB/c	Intraperitoneal	3 doses	*L. donovani*	66% less spleen parasite burden; 71% less liver parasite burden	[145]

**Table 6:** Third-generation vaccines against leishmaniasis over the past 20 years.

Vaccine Name	Parasite Species	Adjuvant	Host	Route	Doses	Challenge	Protection	Reference
pCMV/gp63	*L. major*	-	BALB/c	Intramuscular	Single dose	*L. major*	100× less parasites tan control	[193]
pcDNA3.1-gp63	*L. donovani*	CpG	BALB/c	Intramuscular	2 doses (DNA vaccine) 2 doses (recombinant protein)	*L. donovani* *L. major*	~10^5–6^-fold reduction in the spleen and 10^3^ in the liver Lesion size reduction ~2.5	[195]
pcDNA3.1-gp63	*L. mexicana*	-	BALB/c	Gene gun immunization	2 doses	*L. mexicana*	88% less lesion size	[197]
gp63 amino acid sequence (138–360)	*L. infantum*	-	BALB/c	Intramuscular and subcutaneous	2 doses	*L. infantum*	88% less spleen parasite burden	[199]
pcDNA3-LACK	*L. major*	rIL-12	BALB/c	Subcutaneous	2 doses	*L. major*	~50% less lesion size; 18% less lymph node parasite burden	[200]
pcDNA3.1-LACK	*L. donovani*	-	BALB/c	Intradermal	2 doses	*L. donovani*	Ineffective	[202]
pCI-neo-LACK	*L. chagasi*	-	BALB/c	Intramuscular	2 doses	*L. chagasi*	Ineffective	[203]
pCI-neo-LACK	*L. amazonensis*	-	BALB/c	Intranasal	2 doses	*L. amazonensis*	66% less lesion size; 83% less skin parasite burden	[204]
pCI-neo-LACK	*L. infantum*	-	BALB/c	Intranasal	2 doses	*L. infantum*	90% less liver parasite burden; 85% less spleen parasite burden	[205]
pCI-neo-LACK	*L. infantum*	-	Hamster	Intranasal	2 doses	*L. infantum*	88% less spleen parasite burden; 75 less liver parasite burden.	[206]
pCI-neo-LACK	*L. amazonensis*	Crosslinked chitosan microparticles	BALB/c	Intranasal	2 doses	*L. amazonensis*	51% less lesion size; 99% less skin parasite burden	[208]
MVA-LACK	*L. major*	-	BALB/c	Intradermal and Intraperitoneal	2 doses	*L. major*	90% less lesion size; 80% less lymph node parasite burden	[209]
MVA-LACK	*L. infantum*	-	Hamster	Intramuscular	2 doses	*L. infantum*	66% less liver parasite burden; 83% less bone marrow parasite burden;	[206]
pORT-LACK	*L. infantum*	-	Dogs	Subcutaneous	2 doses	*L. infantum*	25% negative for real-time PCR; 62.5% with only one disease sign	[210]
pPAL-LACK	*L. infantum*	-	Dogs	Intranasal	2 doses	*L. infantum*	60% without disease sign; 92% less bone marrow parasite burden; 80% less spleen parasite burden; 42% less liver parasite burden	[211]
pcDNA3-LiP0	*L. infantum*	-	BALB/c	Intramuscular	2 doses	*L. major*	44% less lesion size; 84% less lymph node parasite burden.	[215]
pcDNA3-LiP0	*L. infantum*	-	Hamster	Intramuscular	3 doses	*L. infantum*	99% less lymph node parasite burden; 99.9% less spleen parasite burden	[216]
pVAX1-P1	*L. donovani*	-	Hamster	Intramuscular	2 doses	*L. donovani*	75.69% less spleen and liver parasite burden	[72]
pVAX1-S20	*-*	-	BALB/c	Intramuscular	3 doses	*-*	Ineffective	Data not published
pcDNA3-TR	*L. major*	-	BALB/c	Subcutaneous	3 doses	*L. major*	22% less lesion size	[218]
MVA-TR	*L. major*	-	BALB/c	Subcutaneous	3 doses	*L. major*	22% less lesion size	[218]
pVAX-1-TR + MVA	*L.* (*V.*) *panamensis*		BALB/c	Intradermal and Intraperitoneal	3 doses	*L.* (*V.*) *panamensis*	77% les skin parasite burden; 90% less lymph node parasite burden	[219]
pcDNA-SOD	*L. donovani*	CpG ODN	BALB/c	Intramuscular	2 doses	*L. major*	33% less lesion size	[224]
pcDNA-GMCSF-SOD	*L. donovani*	CpG ODN	BALB/c	Intramuscular	2 doses	*L. major*	22% less lesion size	[224]
pVAX1-SOD	*L. amazonensis*	-	BALB/c	Intramuscular	3 doses	*L. amazonensis*	42% less lesion size; 71% less skin parasite burden	[225]
pcDNA3-TSA	*L. major*	-	BALB/c	Intramuscular	3 doses	*L. major*	71% less lesion size; 75% less skin parasite burden	[226]
pcDNA3-TSA-LmSTI	*L. major*	-	BALB/c	Intramuscular	3 doses	*L. major*	80% less lesion size	[228]
*Leishmania* multicomponent (10 antigen)	*L. donovani*	-	Dogs	Intramuscular	3 doses	*-*	-	[229]
LEISHDNAVAX	*Leishmania*	-	BALB/c	Intradermal	3 doses	*L. donovani*	94% less liver parasite burden; 91% less spleen parasite burden	[230]
pMOK-Kmp11/-TRYP/-LACK/-GP63	*L. infantum*	-	Dogs	Intradermal	4 doses	*L. infantum*	Ineffective	[231]

## Data Availability

Data are available upon request.
